# Digital divide, skills and perceptions on digitalisation in the European Union—Towards a smart labour market

**DOI:** 10.1371/journal.pone.0232032

**Published:** 2020-04-23

**Authors:** Maria Denisa Vasilescu, Andreea Claudia Serban, Gina Cristina Dimian, Mirela Ionela Aceleanu, Xose Picatoste

**Affiliations:** 1 Department of Statistics and Econometrics, National Scientific Research Institute for Labour and Social Protection, Bucharest University of Economic Studies, Bucharest, Romania; 2 Department of Economics and Economic Policies, Bucharest University of Economic Studies, Bucharest, Romania; 3 Department of Statistics and Econometrics, Bucharest University of Economic Studies, Bucharest, Romania; 4 Department of Economics, EDaSS Research Group on Economic Development and Social Sustainability, University of A Coruna, A Coruña, Spain; Universitat Jaume I, SPAIN

## Abstract

The new technologies, the digitalisation of processes and automation of work will change the manner of doing business, working and living. The effects of digitalisation on the economy, society and quality of life imply significant challenges of the labour market. All the participants will be concerned: authorities, companies and ordinary people. The objective of this research is to analyse the perceptions of the EU citizens about digitalisation and to highlight the differences among specific socio-demographic groups. The analysis is grounded on a composite methodology, comprising several statistical and econometric methods that provide scientific support to achieved conclusions: statistical analysis (with the primary goal to shed light on the EU citizens' perceptions about their digital technology skills), TwoStep Cluster Analysis (TSCA) (with the purpose to identify the ‘digital vulnerable groups’ and then the ‘digital vulnerable countries’ in terms of the exposure to digital divide) and logistic regression (with the main aim to quantify the impact of the relevant factors on citizens’ perceptions about digitalisation). We identified a group of respondents evaluating themselves as having meagre digital skills, very afraid that robots could steal their jobs and with low usage of the internet. They are elderly, with a low level of education, manual workers or not working, with a relatively low level of income and little Internet use. The originality of our approach is given by the fact that we focused on investigating if digital divide leads to the creation of vulnerable groups (citizens and/or countries) and if there are specific patterns in terms of the perception on being skilled in the use of digital technologies in daily life or at work and of the understanding that robots replace human on the labour market. We aim to find relevant factors for the labour market to assume targeted measures that should be taken for a better match of supply and demand on the labour market and for creating a smart labour market. It is highly needed to increase the people's confidence in their skills level and to make the most of digitalisation of the societies. The results show consistent patterns in term of socio-demographic characteristics and perception towards digitalisation. The latter will have a meaningful impact on the economy and the society in the European Union in the next period. That is why a positive attitude towards digitalisation is essential for transforming this relatively new challenge into an excellent opportunity for the future.

## I. Introduction

Digitalisation is one of the most common subjects across all society segments. The technologies associated with this concept are and will amplify each other as they develop. As a result, in recent years, there is an increasing focusing on the impact of the new technologies on the labour market and consequently on people's lives. There are profound changes in all industries that imply changes in occupation structure, as well as in consumption and production patterns [1], [2].

Digitalisation brings major transformation on socio-economic processes [3]. The new technologies will change the manner of doing business, working and living. As a result, it impacts all the participants: authorities/government, companies and citizens/people.

For public authorities, there are changes in the way they interact with citizens and use digital technologies as part as modernisation strategies to create public value [4]. They will focus on the citizens and their needs having as objective their endowment with the appropriate tools for successfully manage the labour market changes. For the government staff, it is also hardly needed to have the tools and the willingness to use the digital technologies for delivering value-added to provided services [5].

For companies, the key element is represented by developing the capacity to respond as fast as possible to their customer needs [6]. Digitalisation brought for them a new battleground for competitive advantage–speed comes first. Also, it will impact the organisational chart (fewer hierarchic level to increase decisional speed), and human resources have to be skills (by adding as compulsory the digital ones) [7].

Citizens are challenged within the digitalisation era. The adaptability becomes of major importance as high skills, and appropriate qualification is required for increasing flexibility to the new changing conditions. Mismanaged by authorities and citizens, it can fuel the vulnerable groups and generate more profound polarisation, group isolation etc.

In this regard, almost two decades ago, the Organization for Economic Co-operation and Development (OECD) has drawn attention to the phenomenon of the digital divide, defined as ‘the gap between individuals, households, businesses and geographic areas at different socio-economic levels with regard both to their opportunities to access information and communication technologies (ICT) and their use of the Internet for a wide variety of activities” [8]. The same study highlighted the main categories of factors that can determine digital divide: a) accessibility of the infrastructure (communication infrastructures, computer availability and Internet access); b) the standard of living (income) and the level of education; c) other factors such as age, gender, racial and linguistic backgrounds and location of the households.

The digital divide is seen as a reflection on the inequalities in society, and it will continue to exist as long as these differences exist [9], [10]. There are mainly two types of gaps: ‘accessibility gaps’ that refer to the differences between urban and rural (urban-rural divide) and ‘user gaps’ that concerns motivation, skills and effective use [11]. Under these circumstances, there is a risk that only a selective group will benefit from the advantages the digitalisation brings [12]. Additionally, there is another significant risk that refers to the problems related to privacy and security, which are growing [13].

The objective of the article is to analyse the EU citizens' perceptions about digitalisation and to highlight the differences between categories with specific socio-demographic characteristics. We have investigated if digital divide leads to the creation of vulnerable groups (citizens and/or countries) and if there are some patterns in terms of the perception on being skilled in the use of digital technologies in daily life or at work and of the understanding that robots replace human on the labour market.

The rest of the paper is organized as follow. The next section comprises the literature review, Section 3 describes the methodology and data used, Section 4 provides results and discussion, and the last section points out concluding remarks.

## II. Theoretical framework

The world is at the beginning of the fourth industrial revolution characterized by developments in robotics, artificial intelligence, internet of things and many other technological advances. Billions of smart devices and machines generate large amounts of data, bringing changes in all areas of activity. Since 1945, computing power increased, on average, by 45% per year and have been improved the computer-controlled automation [14].

The researchers address the transition to the digital economy in numerous studies. According to statistical data, digitalisation brings transformations in all sectors of activity. A growing number of online platforms changes working conditions because people no longer have to be physically present in the workplace. The network and the internet will become the workplace. The studies highlight the advantages of this type of work, related to the flexibility of the lifestyle, the freedom of action, but also the disadvantages, associated with the sacrifice of financial security, the unpredictability of incomes, the need to learn new skills, the increase of the risk of unemployment for specific qualifications [15]. Digitalisation also brings changes in working conditions, as well as in labour legislation, because employment changes considerably, through the use of online platforms, through remote work.

Some authors find the digitalisation as a solution for the problems on the labour market, e.g. the shortage of labour force in specific sectors of activity [16]. Generally, studies show that digitalisation leads to the replacement of jobs that involve repetitive activities and increases the demand for high skills for non- routine tasks. Other authors draw attention to the inequalities that digitalisation generates, between the masses of increasingly isolated low-income workers and the top-of-the-market workers who are in a position to take advantage of the digital instruments [17].

Also, the digital technologies can transform the economic processes, by the following aspects: flexibility of production (due to the processing power), availability of information (digital technologies make data more available), network effects (creating demand-side economies of scale, through social networks, software systems and digital industrial applications), and zero marginal costs (because the digital goods are non-rival and infinitely expandable) [18].

The new technological advances imply the re-skilling of the labour force and changing the world of work, including job substitution, transformation, creation, and lose. The innovation cycle is faster than the changes in the labour market and people's skills [19]. As a result, the imbalances on the labour market growth and are reflected in increasing the duration of unemployment, in long-term unemployment and higher structural unemployment rates [20]. Thus, the current economic environment requires digital knowledge for a large share of total jobs, because digitalisation uses information technology infrastructure and the Internet, as technological support. Digital technologies affect the production, services and all other sectors. Connectivity leads to new dimensions, as electronic devices and microprocessors connect people each other, machines with workers, and machines with machines [21].

Referring to the possibility of replacement the employees by machines, robots and algorithms processes, the risk of automation implies several effects on the labour market. The risk is higher for people with routine jobs and low demand for transversal and social skills and is higher for males and low-skilled workers. The private sector is more exposed to the risk for many reasons. The specialists consider that the cause is related to the failure of providing fast and re-qualification training which highlights the categories exposed to high risk of instability on the labour market and also increases the need for lifelong learning process [22], [23]. Another important reason is that the private sector is adapting faster to the changing economic and technological conditions, and it is more rigorous in judging the failure or success of markets compared to the public sector.

At the European Union level, around one-fifth of employees work from home or are engaged in ICT-based mobile work, meaning they work, from somewhere other than a principal place of work, on a laptop, mobile phone or i-pad. This type of work is mostly used in Northern Countries, Denmark, Sweden, the Netherlands [24].

The implications of digitalisation on the labour market are related to the following aspects [18]:

- the occupational structure is directly and continuously changing, as a result of technological advances (every new technology involves some new way of carrying out a particular process);
- the physical, psychological and environmental requirements and conditions of work are also directly affected by the new information technologies;
- the contractual and social terms of the work, including stability, opportunities for development and payment depend on the institutional framework and labour regulation, which will be affected by the technology;
- how workers and employers organize their relations.
- The vectors of change on the labour market as a result of digitalisation are:
- *automation of work*—the replacement of human by machine. Although machine automation predates even the Industrial Revolution, the use of digital technologies is more automated and allow all kinds of tasks to be automated. Automation transforms the division of labour, because, through automation, the task content of occupations and the importance of some professions will change in respect to others. Even the intellectual non-routine tasks involving creativity, problem-solving and pattern recognition are becoming increasingly open to automation;
- *digitisation of processes*–grows the possibilities of processing, storage and communication of digital information. This framework will increase labour productivity;
- *coordination by platforms*–implies the use of digital networks to coordinate economic transactions algorithmically. These change the employment conditions and allow for the division of labour into tiny tasks [18].

Changes in the labour market resulted from digitalisation, tend to occur more gradually than in consumption, depending on the rate at which young people are entering the market [25]. The main impact of technological change and digitalisation has been an increase in polarization which has affected mostly the middle-level workers for which the income has become more volatile, and uncertainty in the labour market has grown [26].

Some authors [27] have analysed for the United States of America the jobs that can become automated. They have estimated a statistical model of automation potentials and have concluded that 47% of workers in the country will work in automatable occupations in the next 20 years. They considered these jobs automatable occupations, with an estimated automation potential of at least 70%. Other authors have also analysed the degree of automation on the labour market and showed that, on average, 54% of workers in the European Union are working in automatable occupations [28].

It was calculated a potential for automation, focusing on what people do in their jobs rather than relying on occupational descriptions of jobs and it was found that automation potentials are higher in Austria, Germany, Spain, Slovak Republic, United Kingdom and Norway [29]. They find that the share of workers with high automation potentials is highest for unskilled workers and dramatically declines with educational attainment. Also, they show that low-income workers are exposed the most to being potentially automatable. Hence, even though new automation technologies are increasingly capable of performing tasks of highly skilled, the low-skilled workers have functions that are the most exposed to being potentially automatable.

Some studies predict that more and more people (workforce) are *at risk of automation*, which has generated fears of unemployment [22]. Other authors [30] consider that there is no reason for concern from this point of view, because the diffusion of new technologies into the economy is a rather slow process, leaving workers time to adjust, and workers are flexible and adapt. Rather, we have to observe that there have appeared significant structural shifts between occupations and industries, which are accompanied by rising inequality and, weakly, by employment polarization. The main challenge for the future thus is not mass unemployment, but structural changes.

As a result of structural and technological changes, education and continuous training need to be relevant for the current societies and to anticipate the changes they will face for the next period. It will be characterized by an increase in employment in the service sector (mainly in legal and accounting, R&D, advertising) and a decrease in basic manufacturing. The employment rate is expected to grow significantly for highly skilled workers. Also, it is likely some growth for low skilled workers (cleaning, caring, etc.); only medium-skilled workers could face jobs losses (clerks manual workers from agriculture, etc.) [31].

Digitalisation accelerates the transformations of the labour market and accentuates the flexibility in the form of individually tailored temporary work and service contracts, freelance work and multiple jobs [32].

The technological change seems to be associated with a further rise in inequality, as high-skilled, high-wage occupations are on the increase. At the same time, low and medium paid jobs further fall behind. For the prevention of further rising inequality, an important role has education and training according to the needs of the digitized labour market [33]. Digital transformation increases wage inequality to a low extent, the impact of digitalisation on wage inequality remaining rather little [34]. The increasing demand for highly-skilled employees is reflected in an increase in wage inequality. However, the relatively small impact of digitalisation on low-skilled employees prevents a more substantial increase in wage inequality.

The smart labour market will look for workers with digital and entrepreneurial skills, as well as for creativity. Digital skills are fundamental in a smart labour market. The World Economic Forum [35] pointed out that 65% of children entering primary schools today will work in occupations that do not exist yet. These transformations will change the skill sets of present jobs, and it will appear new jobs that require new skills. In this process, it is highlighted the huge role of universities in shaping new skills required by a smart labour market, to provide their students with the adequate skills for future jobs [36]. Large cities with innovation centres and influential universities can offer a favourable environment for the growth of high-tech companies. In this context, the development of ICT infrastructure and entrepreneurial ecosystems play an essential role in the extension of digitalisation [37].

The challenge of skills on the labour market is dual: firstly it should prepare the labour force for the future and, secondly, it should ensure that the current labour force can adjust to changes on the labour market [38]. As a result, lifelong learning will become even more critical in the future [39].

## III. Methodology and data used

### 3.1. Statistical analysis of people’s skills in the use of digital technologies and the perceived impact of robots and artificial intelligence on the labour market

Our analysis is based on The Eurobarometer 87.1 Survey [40]. At EU level, Eurobarometer provides regular monitoring of citizens' attitude, focusing on citizens' perceptions or expectations towards EU action or towards the main challenges the Union is facing. The surveys provide deep insight into the evolution of public opinion on specific issues on a national and a socio-demographic level [41], [42].

The Eurobarometer 87.1 Survey [40] was carried out in March 2017 by Directorate-General for Communication (DG COMM ´Strategic Communication´ Unit) of European Commission, and it focuses on attitude and knowledge regarding the European Parliament and EU, smoking habits, climate change, digital technology and coach services. In our analysis, we have used part D, referring to digital technology, measuring the citizens' perceptions of the current situation and expectations for the future. The people were asked about their perception of the most recent digital technologies (artificial intelligence, the Internet of Things, big data and mobile access to services). These are more and more present in the citizens daily life, and they are offering new products and services (e.g. automated driving, online access to services, connected homes, etc.) The survey provides an extensive view on digital technologies (and it is not limited to technologies like smartphones, tablets, etc.), for this reason, we considered that the perception on the endowment with digital skills is vital for citizens' successful integration/maintaining on the labour market.

We started the analysis by investigating the general perception of the respondents on the impact of recent digital technologies on the economy, on society, and the quality of life. The responses indicated that the perception is positive for all 3 aspects investigated. Specifically, 86.9% of the respondents believe that recent digital technologies have a positive impact on the economy, 75.3% agree with the positive effects on society, and 79.8% of respondents believe that digital technologies lead to an improvement in the quality of life.

One of our main purposes was to analyse the digital technology skills of European Union countries citizens. The question used for this was: “To what extent do you agree or disagree with the following statements regarding your skills in the use of digital technologies: You consider yourself to be sufficiently skilled in the use of digital technologies: i) in daily life; ii) to do your job?”. To evaluate the digital skills, we grouped the answer variants “totally agree” and “tend to agree” and computed the share of respondents that declared having a good level of skills in the use of digital technologies, based on their self-assessment.

**Fig 1:**
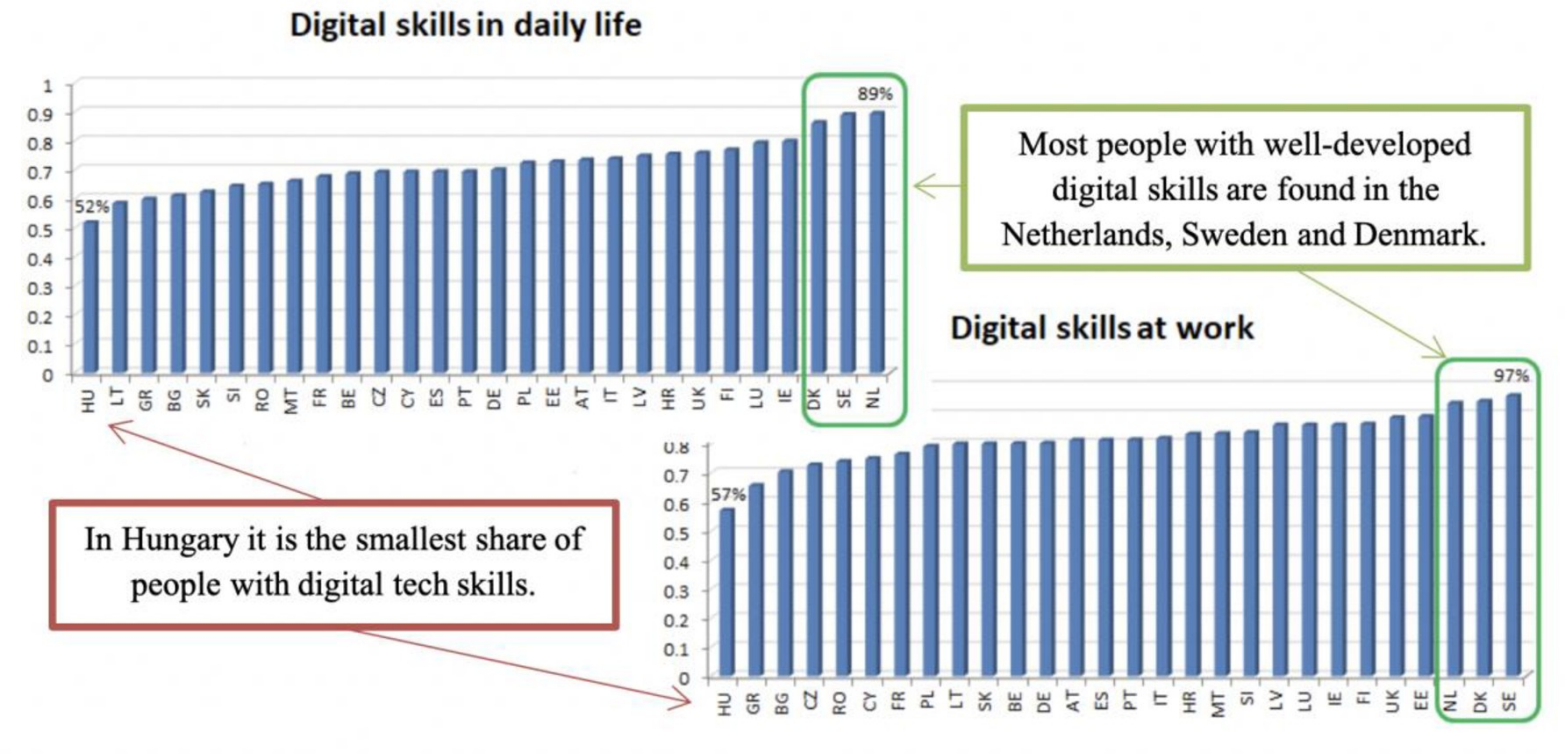
Digital tech skills used in daily life and at work, by country. Source: Authors’ processing using the database of Eurobarometer 87.1 [40].

Following the analysis of the responses, substantial differences were observed between the EU Member States (see Fig 1). Nordic countries stand out, with very high shares of people with good digital skills, used both in daily life, as well as at work. For example, in everyday life, the highest percentage was registered in the Netherlands, where 89% of the respondents stated that they have appropriate skills for using digital technologies. To carry out the activities at work, in Sweden 97% of the respondents said that they have the necessary skills for using digital technologies, being closely followed by Danes (95%) and Dutch (94%). On the opposite side is Hungary, with only 52% of respondents with good digital skills in daily life and 57% sufficiently skilled people in the use of digital technologies at work.

An interesting result was obtained for France, an economically well-developed country, with an advanced level of digital competitiveness– 24^th^ in the world and 11^th^ in Europe [43], being placed in the lower half of the ranking according to the level of digital skills held by the respondents, both for carrying out daily activities, as well as those job-related. Average results for everyday life skills and below average for work-related activities involving digital technologies were also registered in Germany, a very well ranked country in terms of digital competitiveness– 17^th^ in the world and 7^th^ in Europe [43].

We continued the analysis of people’s skills in the use of digital technologies with the investigation of socio-demographic characteristics (Table 1). Gender differences are not substantial, up to 4 percentage points. But it is noteworthy that a higher proportion of men declare that they possess digital skills used in daily life, and a larger share of women is sufficiently skilled to perform their digital tech activities at work.

**Table 1:** Digital tech skills used in daily life and at work, by level of education, occupation, type of community, and difficulties in paying bills (% of respondents that agree having these skills).

		Digital tech skills used in daily life	Digital tech skills used at work
**Gender**	Men	73.00%	87.06%
	Women	69.00%	78.92%
**Age group**	15–24 years	92.70%	86.16%
	25–34 years	91.28%	89.12%
	35–44 years	87.06%	84.49%
	45–54 years	78.92%	78.92%
	55–64 years	64.42%	73.07%
	65 years and older	44.00%	65.92%
**Education**	Low	35.70%	51.50%
	Medium	67.60%	75.20%
	High	85.30%	91.20%
**Occupation**	Students	95.00%	*NA*
	Managers	93.70%	93.60%
	Other white collars	89.00%	88.00%
	Self-employed	83.90%	80.30%
	Manual workers	77.20%	70.10%
	Unemployed	73.80%	*NA*
	House persons	62.30%	*NA*
	Retired	46.40%	*NA*
**Type of community**	Rural area or village	68.20%	78.10%
	Small/middle town	70.30%	81.40%
	Large town	75.70%	84.50%
**Difficulties paying bills**	Most of the time	58.40%	69.20%
	From time to time	68.60%	76.00%
	Almost never/never	73.90%	84.80%

Source: Authors’ processing using the database of Eurobarometer 87.1. [40]

In terms of age, as expected, the ability to use digital technology gradually decreases with age, both in daily life as well as at work. For the digital skills needed at the workplace, the decline is slower, the values recorded for the analysed age groups being closer to each other. Thus, 86% of the interviewed young people (15–24 years) stated that they are digitally skilled to perform their tasks, compared to only 66% of those 65 years and older. The differences are more pronounced for the activities of daily life: 93% of young people can use digital technologies, while only 44% of those 65 and over possess these skills. The more considerable differences recorded between age groups for the skills held in everyday life compared to those used at work may come from the gradual learning of new technologies at work, as part of a structured training activity.

Regarding education, we obtained that the individuals with a low level of education (maximum 15 years of schooling) are the least equipped with the necessary skills to use digital technologies: only 35.7% of them are sufficiently skilled to use digital technologies in daily life, and 51.5% can use them at work. For people with an average level of education (16–19 years of schooling), it was registered that 67.6% agree to have digital tech skills for daily life use, and 75.2% for job-related activities. The highly educated respondents (20 years or more of education) are, of course, the most skilled ones: 85.3% possess the adequate digital skills needed in daily life, and 91.2% are sufficiently qualified to perform digital tech activities at work. Once again we see the overwhelming importance of education—this time, the tendency of massive expansion and development of technology and artificial intelligence must be accompanied by an adequate level of education to benefit everyone and not to deepen inequalities and polarization.

We also analysed people’s digital tech skills according to their occupation. The highest share of people with digital skills used in everyday life was registered for students (95%), even higher than for managers (93.7%) thus indicating the extraordinary openness of young people to technology and their appetite for new. Other white collars (89%), as well as the self-employed (83.9%), also have good digital skills for daily life use, followed by the manual workers (77.2%) and the unemployed (73.8%). It is observed that only 62.3% of the house persons have skills for using digital technologies in everyday life, this result indicating that the people who work or are looking for a job are more connected to the current technological progress. It is worth mentioning that only 46.4% of retirees are sufficiently skilled for using digital technologies in daily life; this result can be due to the combination of two factors: old age and lack of connection with the labour market. Regarding the digital technologies skills used at work, almost all managers (93.6%) are sufficiently skilled to perform their digital tech-related job activities. Next, we see other white collars (88%), the self-employed (80.3%) and the manual workers (70.1%).

There were no major differences between the digital skills of the respondents according to the type of community they live in. Indeed, in the rural area, the share of those with digital skills used both in daily life (68.2%) as well as at work (78.1%) is lower, and those in the big cities are the most skilled, but the differences do not exceed eight percentage points.

Next, we focused on analysing the digital tech skills of the respondents according to their income level, and we used a proxy variable, the difficulties of paying bills because income information is not available in the database. We obtained that of those with relatively low incomes (who frequently face problems paying bills) only 58.4% have digital skills used in daily life, and 69.2% are sufficiently skilled at work. As expected, the highest values were registered for the respondents who do not have difficulties in paying the bills. In essence, 73.9% of them possessed the necessary skills for the use of digital technologies in daily life and 84.8% for using them at work. Low-income households have lower access to technology-related goods and services: they do not always have Internet access, they do not have the financial possibility to purchase high-tech equipment, gadgets or even electronic and home appliances, and therefore their exposure to digital technology is reduced, as well as the possibility of developing such skills.

**Fig 2:**
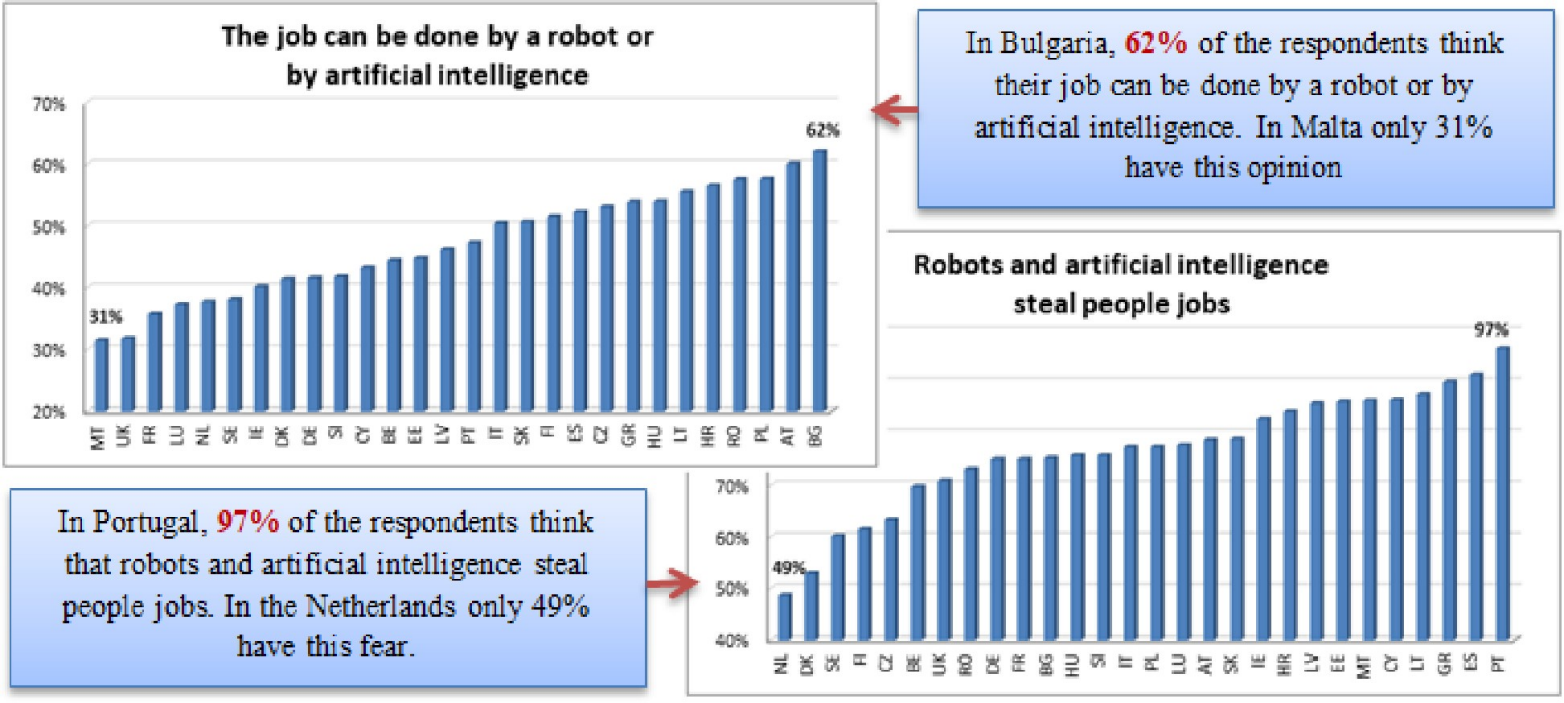
Respondents’ perception of the impact of robots and artificial intelligence on the labour market, by country. Source: Authors’ processing using the database of Eurobarometer 87.1. [40].

The survey refers to robots as machines that assist humans in everyday tasks without permanent guidance and instructions and to artificial intelligence (AI) as systems that can sense, perceive, think and act like humans and behave rationally [40]. The impact of robots and artificial intelligence on the labour market was investigated using two aspects: the respondents’ perception regarding the possibility of a robot to perform their job-related specific tasks (robots substitute low skilled workers) and the opinion that both robots and artificial intelligence steal people's jobs (artificial intelligence needs high skilled workers to be designed and conducted). The perceptions of the EU citizens, by country, can be seen in Fig 2.

Asked if their job can be done by a robot or by using artificial intelligence (totally or partially), 62% of the Bulgarians stated that robots or AI could replace them. Also, large proportions of people who think they can be replaced by robots or AI have been obtained in Austria (60%), Poland (58%) and Romania (57%). At the opposite end is Malta, with only 31% of respondents agreeing that their work can be done by robots or AI, followed closely by the UK (32%) and by France (36%). These results can also be interpreted in the sense of analysing the type of jobs that predominate in the investigated countries—usually repetitive activities, which can be easily automated, are suitable to be assigned to a robot. In contrast, more creative activities, or which involve human interaction and empathy cannot be performed by robots but could be using artificial intelligence.

Regarding the fear that robots and artificial intelligence steal people’s jobs, the only country in which this opinion is not the characteristic of the majority in the Netherlands (49%). Other two countries, Denmark (53%) and Sweden (60%), registered shares relatively low compared to most EU Member States. With percentages between 61 and 70%, we observe another group of countries: Finland (62%), Czech Republic (63%) and Belgium (70%). The larger group of all comes characterized by proportions between 71 and 80% of respondents who consider that robots and artificial intelligence steal people's jobs, 12 countries being in this group: United Kingdom, Romania, Germany, France, Bulgaria, Hungary, Slovenia, Italy, Poland, Luxemburg, Austria and Slovakia. The next category is composed of 8 countries: Ireland, Croatia, Latvia, Estonia, Malta, Cyprus, and Lithuania, being characterised by high percentages of individuals that believe robots steal people’s jobs (between 83% and 90%). Greece, Spain and Portugal are over 90%, meaning that in these countries the opinion that robots and artificial intelligence are stealing jobs is widely widespread. Portugal stands out with 97% of the respondents agreeing with this statement—almost everyone thinks that robots take people’s jobs.

In Table 2, we organised by gender, age, level of education, occupation, type of community, and income level the affirmative answers obtained for the two aspects of the digital technologies investigated in relation to the labour market–respondents’ perception that their job can be done by a robot and the fear that robots and artificial intelligence steal people jobs.

**Table 2:** Respondents’ perception that their job can be done by a robot and the fear that robots and artificial intelligence steal people jobs (% of respondents that agree).

		The job can be done by a robot	Robots and AI steal jobs
**Gender**	Men	50.1%	73.0%
	Women	45.2%	78.9%
**Age group**	15–24 years	55.6%	73.9%
	25–34 years	51.9%	74.2%
	35–44 years	48.4%	73.6%
	45–54 years	47.2%	76.1%
	55–64 years	42.9%	77.5%
	65 years and older	33.9%	78.7%
**Education**	Low	48.1%	87.5%
	Medium	49.7%	80.2%
	High	45.2%	67.6%
**Occupation**	Students	*NA*	71.1%
	Managers	41.9%	65.2%
	Other white collars	50.8%	74.1%
	Self-employed	41.5%	70.5%
	Manual workers	51.0%	79.0%
	Unemployed	*NA*	81.2%
	House persons	*NA*	84.9%
	Retired	*NA*	78.7%
**Type of community**	Rural area or village	45.5%	78.9%
	Small/middle town	47.2%	75.6%
	Large town	50.2%	73.9%
**Difficulties paying bills**	Most of the time	51.0%	84.9%
	From time to time	52.8%	81.1%
	Almost never/never	45.1%	73.1%

Source: Authors’ processing using the database of Eurobarometer 87.1. [40]

It is interesting to note that over half of men consider that their work can be carried out by a robot (50.1%) compared to 45.2% of women with a similar opinion. It is possible for men to be more involved in the production, assembly, repetitive activities, which could be easily automated and performed by a robot, and women to have jobs instead of in creative domains and fields that require human interaction. On the other hand, women are more scared than men that robots steal people's jobs, 78.9% having this opinion.

With age, there is a downward trend in the share of respondents who believe that robots can do their job. The highest percentage was registered among young people (15–24 years), 55.6% of them considering that they can be replaced by artificial intelligence at work. At the other end of the distribution are the elderly, only 33.9% of those aged 65 or over having the opinion that the activities carried out at their workplace can be performed by robots. These differences by age groups can be explained by the much higher exposure of young people to new technologies, which makes them more aware of the rapid development of digitalisation and its massive involvement in the economy, in society and people's lives.

Regarding the fear that robots steal people's jobs, no significant differences have been obtained between the age groups, the percentage of those who have this opinion ranging between 73.6% and 78.7%. The minimum value was registered for the age group 35–44 years, which generally contains the persons at the peak of their career, productive and confident workers. The maximum amount was observed among the elderly, an aspect that can be explained by their more considerable reluctance to change and that the fear is commonly exacerbated by what they do not know or master very well.

The results obtained by level of education indicated that 49.7% of the respondents with a medium level of education consider that their job can be done by robots, 48.1% of those with a relatively low level of education have this opinion and 45.2% of the respondents with higher education believe that they can be replaced by robots at work. The fear that robots and artificial intelligence steal people’s job is more widespread among those with a low level of education: 87.5% have this concern. We can also notice a significant difference compared to the higher education graduates, of more than 20 percentage points. This result may indicate that those with a low level of education feel more vulnerable to losing their job due to new technologies.

The analysis by occupation groups comes with a confirmation of the theory that manual and repetitive tasks are more likely to be performed by robots. The results indicate that 51% of the manual workers consider that their work can be done by a robot, the highest percentage recorded. At the opposite end, we find the self-employed, with only 41.5% who believe that their job can be done by a robot or by artificial intelligence. Answers regarding the fear that robots steal people's jobs have indicated that 84.9% of house persons agree with that statement, followed closely by the unemployed (81.2%). These high percentages recorded for the two categories may indicate their difficulty in finding a job and their belief that digital technology and artificial intelligence come as an additional threat to their chances of integrating into the labour market. In correlation with their low digital skills, technological progress will deepen the differences between them and other social categories, significantly increasing their risk of exclusion and poverty. Only 65.2% of managers consider that robots steal people's jobs, the lowest percentage recorded. This result is a sign of an opinion closer to reality and not a response based on emotions: although digital technologies will have a substantial impact on the labour market, there are many jobs that will not be taken over by robots. Also, some jobs will disappear, but others will be created, so the role of people in the labour market will remain an important one.

When analysing the answers according to the type of community the respondents live in, we noticed a slightly increased percentage of people thinking that robots can replace them at work in the large towns (50.2%) compared to those living in small or medium cities (47.2%) or the rural area (45.5%). Once again, more significant contact with digital technologies increases the awareness of the changes they imply on the labour market. Moreover, the analysis of the opinion that robots steal people's jobs illustrates an inverse hierarchy: a higher share of villagers are worried that artificial intelligence leaves people without jobs (78.9%), compared to the inhabitants of big cities (73.9%).

More than half of the respondents who stated that they face difficulties paying the bills from time to time or most of the time believe that robots can do their jobs, as opposed to those who do not have problems with paying bills, of which 45.1% have this opinion. Regarding the fear that robots and artificial intelligence steal people's jobs, 84.9% of the respondents with financial difficulties, 81.1% of those having difficulties for paying bills from time to time, and 73.1% of the respondents that always can afford to pay their bills agree with this statement.

An interesting aspect is that of those who say that their work cannot be done by a robot; 72.6% agree that robots steal people's jobs.

### 3.2. Descriptive statistics

The list of the variables used in the logistic regression, as well as their description, is presented in Table 3.

**Table 3:** Description of the variables used in the logistic regression.

*Category*	*Variable*	*Type*	*Range*	*Mean*	*Std*. *dev*.
Dependent variables	Respondent digital tech skills in daily life (Model 1)	binary	0–1	0.71	0.453
	Respondent digital tech skills to do job (Model 2)	binary	0–1	0.81	0.390
	Respondent’s opinion that robots and AI steal peoples' jobs (Model 3)	binary	0–1	0.76	0.426
	Respondent’s opinion that his current job could be done by a robot or by AI in the future (Model 4)	binary	0–1	0.48	0.499
Socio-demographic variables	The respondent’s gender	binary	0–1	0.45	0.498
	The respondent’s age	categorical	1–4	3.09	1.008
	Education	categorical	1–3	2.22	0.696
	Type of community	categorical	1–3	1.95	0.775
	Marital status	binary	0–1	0.64	0.479
	The social class of the respondent	categorical	1–5	2.35	0.988
	Respondent’s occupation (for Model 1)	categorical	1–3	2.46	0.611
	Respondent’s occupation (for Models 2, 3 and 4)	categorical	1–6	3.45	1.446
	Difficulties paying bills–last year	categorical	1–3	2.55	0.663
	Internet use	categorical	1–4	3.2	1.242
Variables describing personal opinions	Recent digital technologies—impact on economy	binary	0–1	0.87	0.337
	Recent digital technologies—impact on society	binary	0–1	0.75	0.431
	Recent digital technologies—impact on daily life	binary	0–1	0.80	0.401
	Robots—general appraisal	binary	0–1	0.67	0.471
	Read about artificial intelligence in the last 12 months	binary	0–1	0.47	0.499

### 3.3. Research methodology and hypotheses

The purpose of the empirical analysis is twofold: 1) to examine if digital divide could lead to the creation of ‘digital vulnerable groups’ and more than that of ‘digital vulnerable countries’ and 2) to analyse the people’s perception about digital skills used in daily life, as well as at work and also the opinion about the impact of robots on their jobs and, generally, on the labour market. Hence, we have formulated four research hypotheses:

H1. The digital divide could lead to the creation of ‘digital vulnerable groups’ and more than that of ‘digital vulnerable countries’.
H2. The effective use of new technologies (in daily life as well as at work) depends on perceptions and skills, which in their turn are mainly determined by the level of education and income.
H3. The general perception of EU citizens about the digitalisation is a positive one, but some categories feel insufficiently prepared for the assimilation of new technologies, especially in their workplace.
H4.People’s perception of robots is generally a positive one, but there are significant concerns especially regarding the impact of digitalisation on the labour market and jobs

To address these hypotheses, we have chosen two suitable econometric techniques: TwoStep Cluster Analysis (TSCA) (H1) and Logistic Regression (H2-H4).

#### A. Two Step Cluster Analysis (TSCA)

The first hypothesis of our research states that certain groups of people are more exposed to digital divide than others and more prone to become vulnerable (i.e. to be left behind) in terms of the attitude towards digitalisation, digital skills and use of the internet. More than that, the countries with large shares of citizens with reduced digital skills and use of the information and communication technology are more likely to experience vast social inequalities and development gaps compared to the other countries.

To test the first hypothesis H1 of our research, we have chosen to apply cluster analysis with the aim to create homogenous groups of respondents regarding the exposure to the digital divide and to identify the ‘digital vulnerable groups’ and then the ‘digital vulnerable countries’. TwoStep Cluster Analysis (TSCA) has been selected due to the ***main advantages*** that it offers. In essence, it can be applied both on *continuous and categorical variables*, and the *optimal number of clusters* can be determined automatically based on informational criteria. Besides, TSCA efficiently analyzes *large data sets*. Hierarchical and K-Means Cluster, classical cluster analysis methods, use hierarchical or partitioning algorithms for classification. Still, each has some drawbacks: Hierarchical Cluster Analysis is usually used for analyzing small data sets, while K-Means Cluster Analysis can be applied only on continuous variables. As regards TSCA, two limitations are highlighted in the literature: the way it approaches the missing values and the influence of the order of the registrations on the cluster analysis results.

TSCA uses an algorithm that allows grouping a large number of observations into natural groups. The clustering process is based on a similarity criterion that implies distances computation. In the case of categorical variables, the distance measurement can be obtained by calculating the *log-likelihood measure* (the probability distributions of the variables with the aim to capture the probabilities of being part of a particular cluster). Certain ***assumptions*** must be met such that likelihood distance measure to give the best results: the normal distribution of the continuous variables, respectively the multinomial distribution of the categorical variables. A third assumption is the independence of the variables included in the cluster analysis. We have verified if the categorical variables included in the cluster analysis meet the two aforementioned assumptions (multinomial distribution and independence) using the goodness of fit test for multinomial distribution and independent samples Kruskal-Wallis test. The results confirmed our initial supposition that our variables might not pass these tests. In order to obtain the best possible results, we have chosen to include in the cluster analysis the least correlated variables in terms of the Kendall's_tau_b coefficient. However, internal testing made by IBM Knowledge Center demonstrated that TSCA ‘procedure is reasonably robust to violations of both the assumption of independence and the distributional assumptions’ [44].

As its name suggests, TSCA involves the formation of groups (clusters) in two stages (see Eqs A.1–A.6):

a) A *pre-clustering stage*, consisting of the construction of Cluster Features Tree (CF), which contain cluster centres. These appear in the form of leaf nodes that synthesize the information about the analyzed cases regarding the variables considered.

The log-likelihood distance between a cluster *i* and cluster *s* can be defined as follows [45]:

$$d(i,s)=\xi_{i}+\xi_{s}-\xi_{\langle i,s\rangle }$$ (A.1)

The dispersion within such a cluster incorporates two types of variances: the variance of the continuous variables and the entropy (a measure for the variance of the categorical variables).

$$\xi_{v}=-n_{v}\left(\sum_{j=1}^{p}\frac{1}{2}\log({\hat{\sigma }}_{vj}^{2}+{\hat{\sigma }}_{j}^{2})-\sum_{j=1}^{q}\sum_{l=1}^{m_{j}}{\hat{\pi }}_{vjl}\log({\hat{\pi }}_{vjl})\right)\;\mathrm{where}\;{\hat{\pi }}_{vjl}=\frac{n_{vjl}}{n_{v}}$$ (A.2)

And *ξ*_*v*_ is a type of dispersion within-cluster *v*, *n*_*v*_ is the total number of observations in cluster *v* (*v = i*,*s*, *<i*,*s>*), *p* is the total number of continuous variables *x*_*j*_ (*j = 1*,*2*,*…*,*p*), *q* is the total number of categorical variables *a*_*j*_ (*j = 1*,*2*,*…*,*q*), *m*_*j*_ is the total number of categories of the variable *a*_*j*_ (*l = 1*,*2*,*…*, *m*_*j*_), ${\hat{\sigma }}_{j}^{2}$ is the estimated variance of the variable *j* in total observations, ${\hat{\sigma }}_{vj}^{2}$ is the estimated variance of variable *j* in cluster v, ${\hat{\pi }}_{vjl}$ are the estimated probabilities of distribution of the categorical variables *a*_*j*_ in cluster v, *n*_*vjl*_ is the number of observations for variable *j* that take *l* category within-cluster *v* [46].

When only categorical variables are included in the TSCA, the entropy within *k* clusters is [45]:

$$l_{k}=\sum_{v=1}^{k}\xi_{v}$$ (A.3)

b) The *clustering stage* itself leads to a set of solutions by applying the agglomerative clustering algorithm. Of these, the best solution can be obtained using an informational criterion (Schwarz, Bayesian Criterion-BIC, or Akaike Information Criterion-AIC) [45].

$$AIC_{k}=-2l_{k}+2r_{k}$$ (A.4)

and

$$BIC_{k}=-2l_{k}+r_{k}\log n$$ (A.5)

where the number of independet parameters *r*_*k*_ is given by:

$$r_{k}=k\left\{2p+\sum_{j=1}^{q}\left(m_{j}-1\right)\right\}.$$ (A.6)

In this step, two measures are computed: the ratio of BIC (or AIC) changes used to find the maximum number of clusters and the rate of distance measures used to find the optimal number of clusters.

#### B. Logistic regression

There are many ways to evaluate inequalities, the most common techniques being the Gini index, the Lorenz curve or Theil index [47]. But in this case, the database did not allow their use, having rather qualitative variables, so we turned our attention to the logistic regression method.

***The logistic regression model*** (Eqs B.1–B.11) is used when the dependent variable is binary. A binary variable is a qualitative variable representing the presence or absence of a probabilistic event. The presence of the event is generally coded 1, and the absence it is coded 0 [48].

To study the probability that *y*_*i*_ = 1, we have at our disposal a set of *k* explanatory variables *x*_*i*1_, *x*_*i*2_,…,*x*_*ik*_, that can be expressed under the form of a vector *X*_*i*_. The probability model can be written:

$$P(y_{i}=1|X_{i})=F(\beta_{0}+\beta_{1}x_{i1}+\beta_{2}x_{i2}+\cdots +\beta_{k}x_{ik})=F(X_{i}\beta )$$ (B.1)

where *P*(*y*_*i*_ = 1|*X*_*i*_) represents the conditional probability that *y*_*i*_ equals 1 given the characteristics *x*_*i*1_, *x*_*i*2_,…,*x*_*ik*_. *β* is a vector consisting of *k + 1* parameters. *F*(∙) is the distribution function of *β*_0_+*β*_1_*x*_*i*1_+*β*_2_*x*_*i*2_+⋯+*β*_*k*_*x*_*ik*_. The properties of the function *F*(∙)are such that for any variable z, lim_*z*→−∞_*F*(*z*) = 0 and lim_*z*→+∞_*F*(*z*) = 1. *F*(∙)is therefore a positive continuous function taking values between 0 and 1.

Based on these previous properties, the discrete choice model is written as follows:

$$\left\{ \begin{array}{l} P(y_{i}=1)=F(X_{i}\beta )\\ P(y_{i}=0)=1-F(X_{i}\beta ) \end{array}\right.$$ (B.2)

The general form of the model is:

$$y_{i}=F(X_{i}\beta )+\varepsilon_{i}$$ (B.3)

One of the most popular binary response models is the logit model [49]. The logit model corresponds to the choice of a logistics distribution function *F*(∙), defined for a variable *z* as follows:

$$F(z)=\frac{e^{z}}{1+e^{z}}$$ (B.4)

The distribution function of the logistic law can also be deduced from the density function:

$$F(z)=\int_{-\infty }^{z}\phi (t)dt=\phi (z)$$ (B.5)

Where 𝜙 (𝑡) is the density function of the logistic law defined as follows

$$\phi (t)=\frac{e^{z}}{1+e^{z}}-\frac{e^{2z}}{{\left(1+e^{z}\right)}^{2}}$$ (B.6)

The functions *F*(∙)and 𝜙 (z) are defined so that *lim*_*z*→−∞_*F*(*z*) = *lim*_*z*→−∞_*ϕ*(*z*) = 0 and *lim*_*z*→+∞_*F*(*z*) = *lim*_*z*→+∞_*ϕ*(*z*) = 1.

$$\mathrm{Moreover},\;\phi (t)=F^{\prime }(t)=f(t)=\frac{dF(z)}{dz}.$$ (B.7)

Given these properties, the model becomes:

$$P(y_{i}=1|X_{i})=F(X_{i}\beta )=\frac{e^{z}}{1+e^{-X_{i}\beta }}$$ (B.8)

The coefficients obtained from the estimation of a logit model have a very interesting interpretation. This is the odds ratio. We know that in the logit model:

$$\frac{P(y_{i}=1)}{1-P(y_{i}=1)}=e^{X_{i}\beta }$$ (B.9)

This equality represents the odd of event 1. By taking this equality in logarithm, we find the quantity called log-odds:

$$ln\left(\frac{P(y_{i}=1)}{1-P(y_{i}=1)}\right)=X_{i}\beta$$ (B.10)

In general, to calculate the odds ratios, we calculate the exponential of the coefficient 𝛽. So, we have:

$$OR=e^{\beta }$$ (B.11)

The odds ratios are prefered when interpreting the results of logistic regression as they represent the effect of an explanatory variable X on the likelihood that the analysed outcome will occur.

In our analysis we are interested in obtaining the odds ratios to see if an individual is more likely to have, for example, the digital skills needed in daily life (model 1) depending on specific socio-demographic characteristics or personal opinions. The odds ratios also help in analysing inequalities: for example, when investigating gender disparities, we will be able to see if men (or by contrary, women) have more chances to be digitaly skilled. Moreover, these results are quantitative, indicating to what extent men (or women) are more likely to have the necessary digital skills compared to the reference category.

## IV. Results and discussion

### 4.1. TwoStep Cluster Analysis results

*TwoStep Cluster Analysis* (TSCA) has been applied with the aim to create homogenous groups of people in terms of three aspects related to digitalisation: *the attitude* towards digitalisation (with emphasis on the concerns about the impact of the robots on the labour markets), the perception of EU citizens on their own *digital skills* (looking for the digitally vulnerable groups) and the actual *use of technology* (use of the Internet).

The first step was to analyse the solution with the maximum number of clusters, looking for those who are very different from the others. Next, from the optimal number of clusters, we selected one bunch that we identified with ‘digital vulnerable group’ of respondents.

In a second stage, the results of the clustering were evaluated from the point of view of the main characteristics of the persons who were included in the clusters: *gender* (female, male), *age groups* (15–24 years; 25–39 years, 40–54 years, 55 years and older), *occupations* (self-employed, managers, other white collars, manual workers, house persons, unemployed, retired, students), level of *education* (low, medium and high), *difficulties paying bills* (most of the time, from time to time, almost never/never).

The central hypothesis was that digitalisation and, more than that, the digital divide, could lead to the formation of some ‘digital vulnerable groups’ and some ‘digital vulnerable countries’. We identify ‘digital vulnerable group’ with a cluster of citizens having low digital skills, very afraid of the transformations that digitalisation could bring into their lives or at their jobs and reduced use of the new information and communication technology. Specific social categories are more likely to be ‘digital vulnerable’: women, employees over 50 years, employees with a low level of education, people with occupations that require low skills, the population from underdeveloped areas. ‘Digital vulnerable countries’ are those with large shares of citizens with low digital skills and less accessibility to new technologies.

As the descriptive analysis has highlighted, the general opinion of the EU population about digitalisation and its impact on economy, society and daily life is in general positive. Also, the respondents in the surveyed sample have a good and very good perception of their own digital skills and consider that the robots could not replace them in the future in the workplace. However, fears that robots might steal their jobs or that more jobs will disappear than jobs will be created in the future due to digitalisation, are also very high. Thus, the attitude towards digitalisation is quite a controversial one, and public and private policies should take into account both citizens’ concerns and the existence of some groups more vulnerable than others in terms of the access on the labour market. In the digitalisation era and in a period of deep demographic transformations, like ageing, everyone should take better care of what we call ‘human capital’ (providing high-quality education for the young generation and lifelong learning opportunities for older generations) [50].

Starting from these considerations, a second step in investigating the opinions of the EU population about digitalisation was to examine the existence of some perception patterns or, in other words, whether homogeneous groups of people can be obtained based on their opinions on three critical aspects of the relationship with digitalisation: attitude towards digitization/robotization, perception on own skills and effective use of ICT.

We intended to examine at this stage of the analysis what are the perception patterns, focusing on the implications of digitalisation on the labour market. Moreover, we aimed to verify the existence of what we named 'digital vulnerable' social groups. We assumed that people who have a very good perception of their skills to use ICT / the Internet intensively and to be less fearful about the possible job loss in favour of robots. In this group should be found the so-called ‘white-collar’ occupations, made by young people, rather men (fewer females) with a high level of education and a level of income ensuring a decent standard of living. We supposed that these people come from developed economies (countries that have made significant investments in digital infrastructure as well as in digital education).

At the opposite pole, we assumed that there should be a group of people who are less confident in their skills and convinced that robots can replace them in the future in the workplace. Their fears about losing their jobs should be high. Both women and men of older age with a medium or low level of education, with occupations easily replaceable by robots (as manual workers or house persons) and with a low level of income could be part of this group.

Three items (categorical variables) have been included in the Cluster Analysis:

**I1.** Robots steal jobs (1 = Totally disagree, 2 = Tend to disagree, 3 = Tend to agree, 4 = Totally agree)
**I2.** Respondent digital tech skills to do the job (1 = Totally disagree, 2 = Tend to disagree, 3 = Tend to agree, 4 = Totally agree)
**I3.** Internet use (total) (1 = No Internet access at all, 2 = Never, 3 = Often/ sometimes, 4 = Everyday)

Based on Schwarz's Bayesian Criterion-BIC, the maximum number of clusters was determined, 15 in this case. The ratios of distance measures have been used to establish the final number of clusters, respectively, 4 (Table 4).

**Table 4:** Number of clusters according to schwarz’s bayesian criterion (bic) and ratio distance measures.

Auto-Clustering				
Number of Clusters	Schwarz's Bayesian Criterion (BIC)	BIC Change^a^	Ratio of BIC Changes^b^	Ratio of Distance Measures^c^
1	73712.122			
2	59526.843	-14185.279	1.000	1.170
3	47415.911	-12110.932	.854	1.341
4	38405.179	-9010.732	.635	1.785
5	33395.861	-5009.318	.353	1.079
6	28760.288	-4635.573	.327	1.033
7	24274.982	-4485.306	.316	1.172
8	20460.740	-3814.242	.269	1.435
9	17829.406	-2631.334	.185	1.254
10	15748.099	-2081.307	.147	1.186
11	14005.764	-1742.335	.123	1.193
12	12558.788	-1446.976	.102	1.183
13	11348.644	-1210.144	.085	1.284
14	10424.909	-923.734	.065	1.034
15	9534.672	-890.237	.063	1.184

a. The changes are from the previous number of clusters in the table.

b. The ratios of changes are relative to the change for the two cluster solution.

c. The ratios of distance measures are based on the current number of clusters against the previous number of clusters.

Source: Authors' computations [40], using *IBM SPSS Statistics 21*

**Fig 3:**
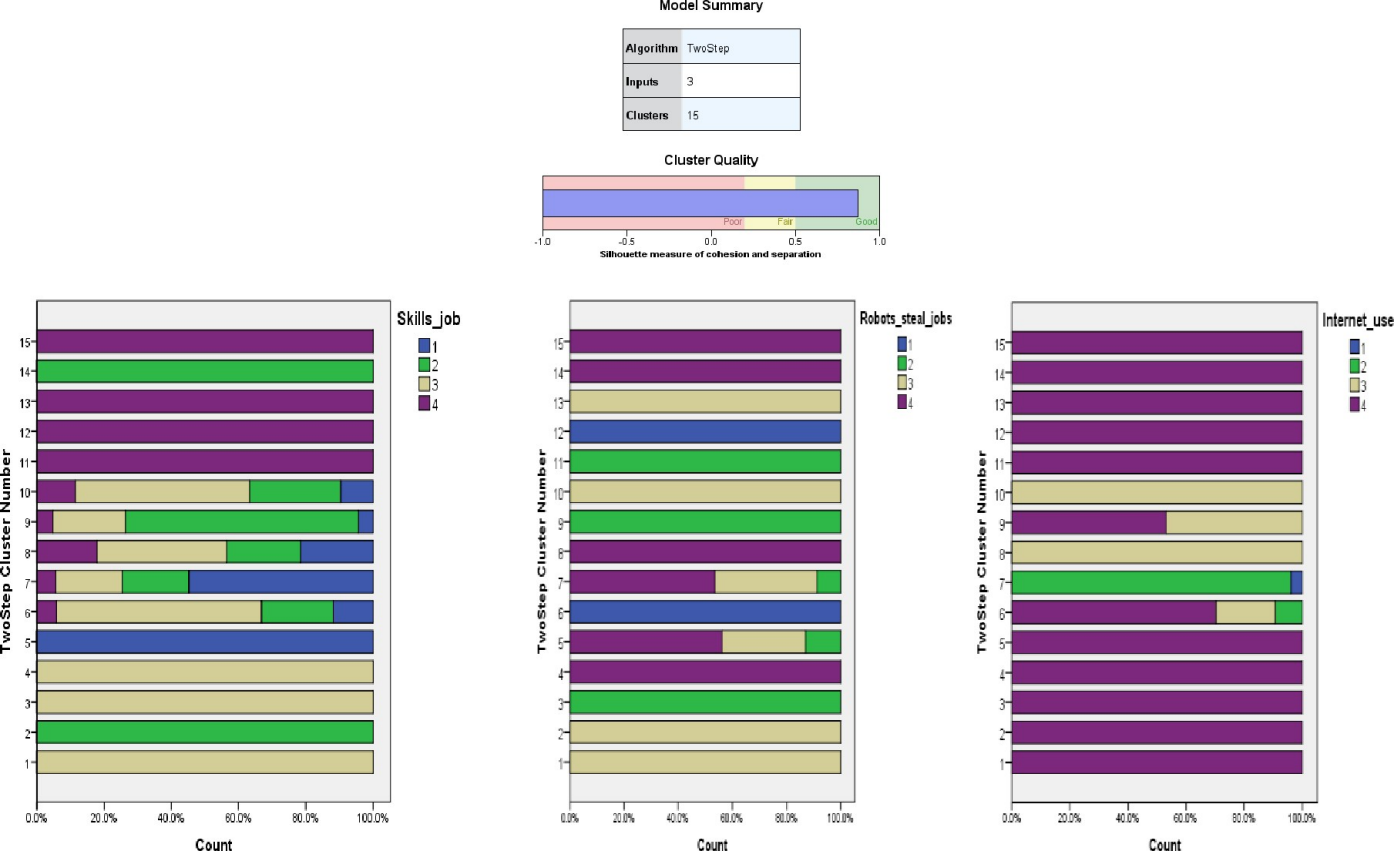
Main features of the 15 clusters. Source: Authors' computations [40], using IBM SPSS Statistics 21.

For the beginning, we have identified the 15 clusters and analysed their features: common trends and main differences. For the model with 15 clusters, silhouette measure of cohesion and separation was 0.870, very closed to the maximum value of 1.We had applied the Kruskal-Wallis test to verify the independence of the clusters in relation to their components, but also to make pairwise comparisons. The 15 clusters prove to be independent, and the pairwise comparisons demonstrated that two clusters are the most different: cluster 7 with over 50% of the respondents having very low digital skills, very afraid that robots could steal their jobs and with low usage of the internet. At the opposite end, there is cluster 12, with all the respondents having very high digital skills, not at all afraid of robots stealing their jobs and very high usage of internet (Fig 3).

**Fig 4:**
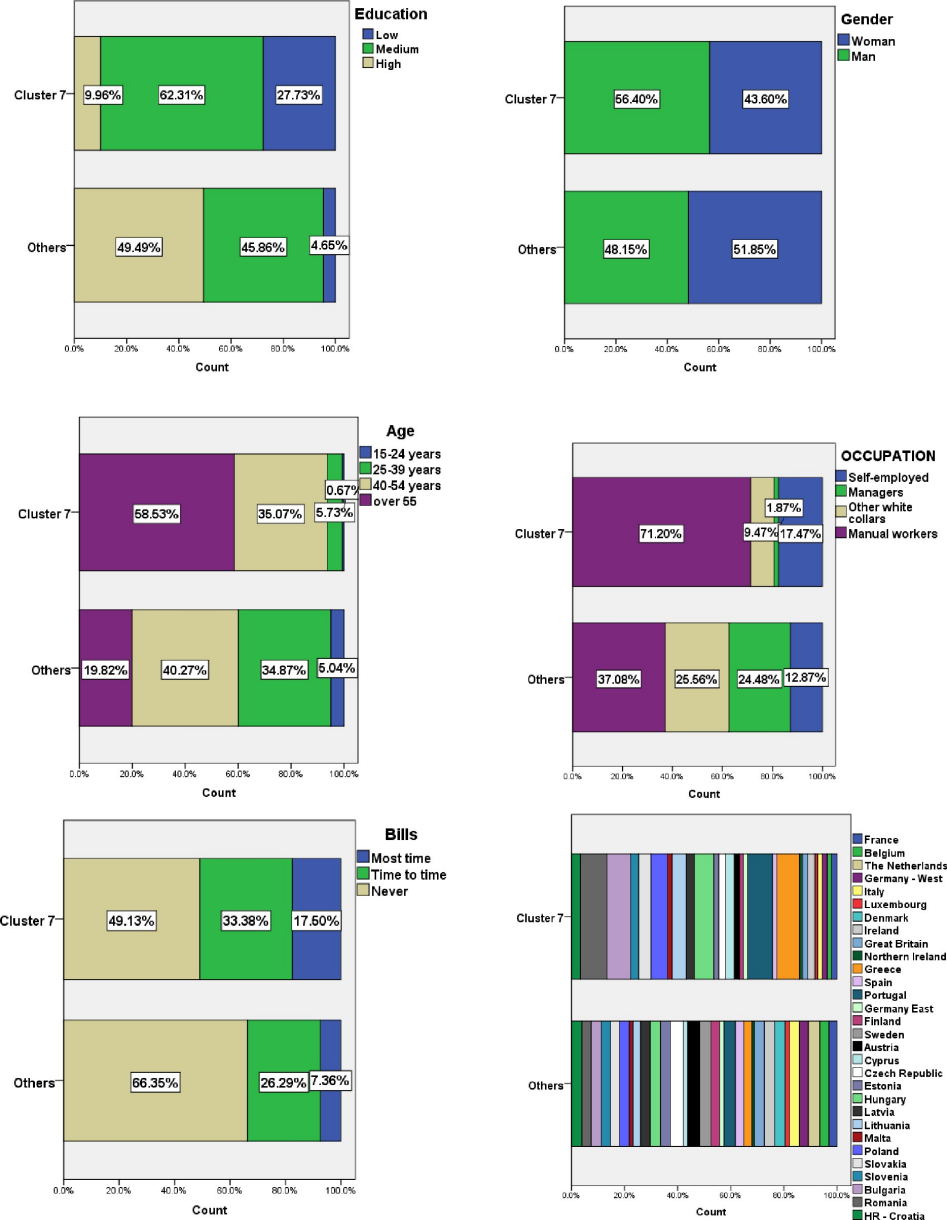
Main features of the cluster number 7. Source: Authors' computations [40], using IBM SPSS Statistics 21.

We have identified cluster 7 with the most digital vulnerable groups of respondents: 90% of the respondents in this cluster have low or medium education level, 56% are women, approximately 60% have over 55 years, 71% are manual workers and a half of them have problems in paying bills. Almost 50% of this cluster is composed of respondents coming from countries like: Romania, Bulgaria, Hungary, Portugal and Greece. Around 50% of cluster 12 is composed of respondents coming from three countries: Sweden, Denmark and the Netherlands (Fig 4).

The optimal number of clusters have been established based on the ratios of distance measures at four.

**Fig 5:**
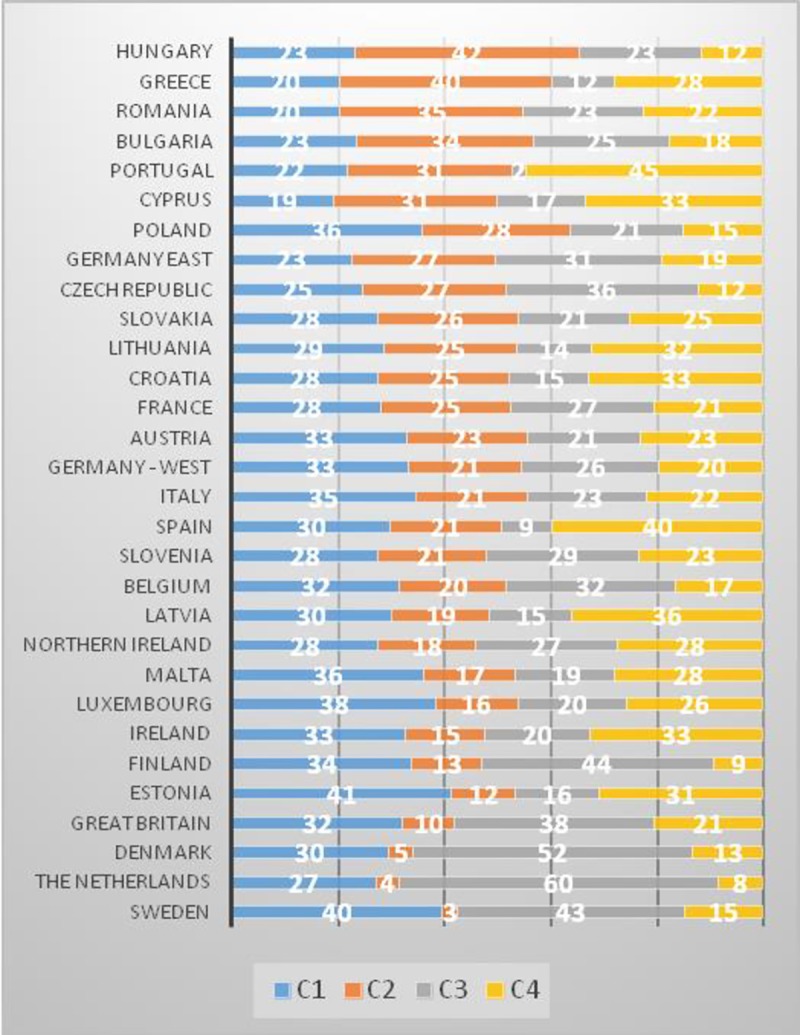
Respondents’ distribution by clusters and countries (%). Source: Authors' computations [40].

*Cluster number 1* is the largest in terms of the included observations (3675 respondents, representing 29.3% of total), followed by *cluster 3* (3229 respondents, representing 25.7% of total) and *cluster 4* (2899 respondents, representing 23.1% of total). The rest of the observations grouped in cluster *number 2* (2753 respondents, representing 21.9% of total). As regards the countries from which the respondents come from, it can be seen that Cluster 1 includes a large part of the population of countries such as Estonia, Sweden, Luxembourg, Malta, Poland and Italy (with shares between 35% and 41% of total). Large parts of the population from the Netherlands, Denmark, Finland, Sweden, the United Kingdom and Czech Republic grouped in cluster 3 (with shares between 38% and 60% of total). In cluster 4, we found a large part of the population from countries such as Portugal, Spain, Latvia, Cyprus, Croatia (with shares between 33% and 45% of total). In cluster 2, a large part of the population comes from countries such as: Hungary, Greece, Romania and Bulgaria (Fig 5).

**Fig 6:**
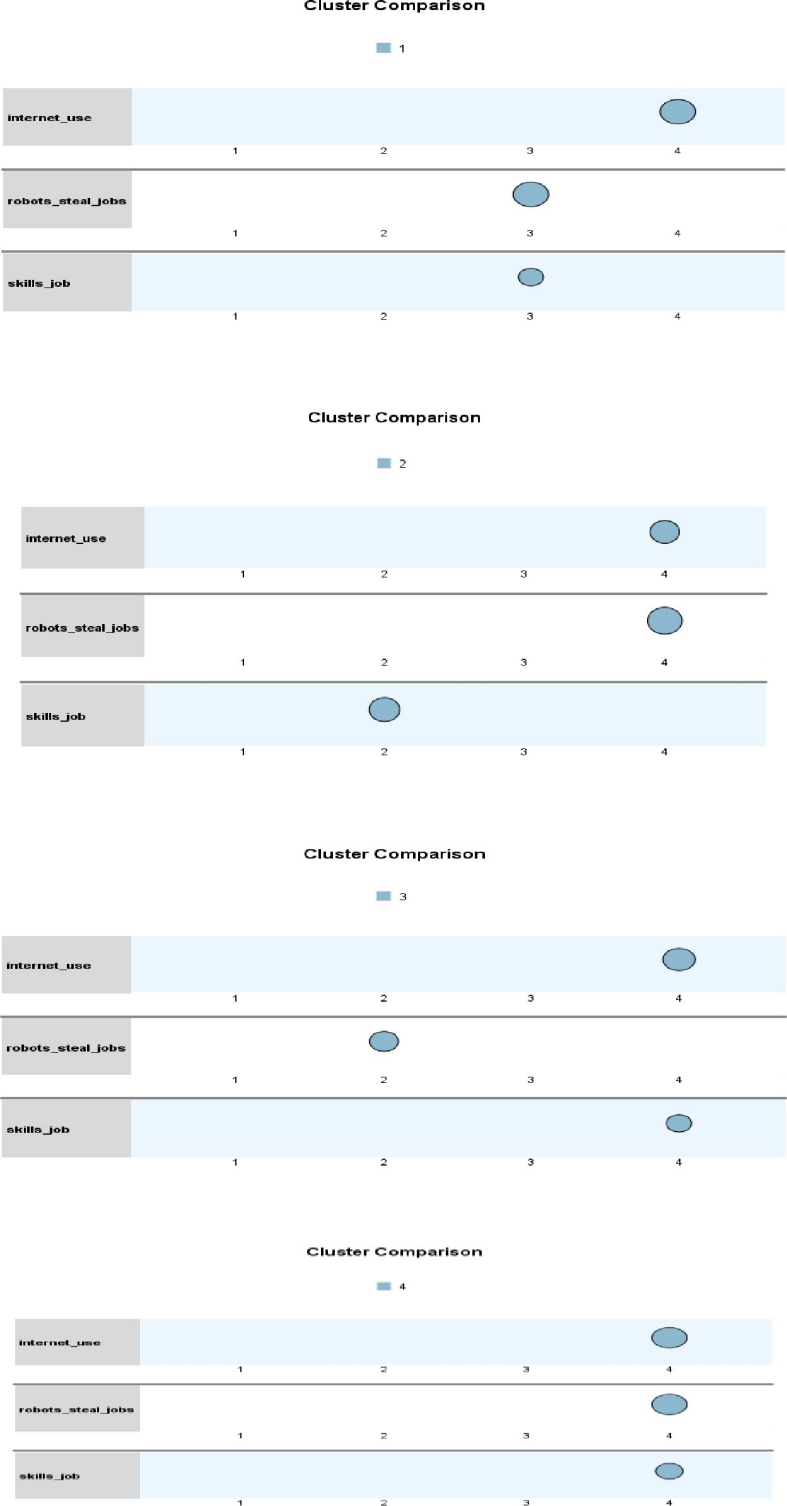
Main features of the four clusters. Source: Authors' computations [40], using IBM SPSS Statistics 21.

*Cluster number 1* includes the respondents in the sample that *tend to agree* both with the statement that they are sufficiently skilled in the use of digital technologies to do their job and that the robots steal people jobs. This group stated that they are using Internet every day. On the assertion that robots steal jobs, the most frequent category selected by the respondents in this cluster was 3 (100% of them tend to agree with the statement). The same answer was most frequent when the respondents were asked about having the necessary digital skills to do their job (50.5% of them tend to agree with the affirmation). In this cluster, the most frequent category registered for the item ‘Internet use’ was 4 (100% of the cluster respondents stated that they are using the Internet every day). We assessed this cluster as that of the ‘*Internet users*, *confident in their digital skills*, *but fearful that robots might steal their jobs’* (Fig 6).

The respondents who *totally agree* with the claim that they have the necessary digital skills to do the job and who *tend to disagree* that robots steal jobs grouped in *cluster 3*, second in terms of the size after cluster number 1. This group of people, like the first, states that it uses the Internet every day. 94.9% of the respondents in this group chose category 4 (every day) for the item regarding the use of the Internet. 76.2% chose category 2 (tend to disagree) to the affirmation regarding robots that steal jobs, and 57.7% chose category 4 (totally agree) to the affirmation regarding own digital skills to do job. This cluster can be seen as the group of ‘*Internet users*, *very confident in their digital skills and only a little afraid of the robotization of jobs’* (Fig 6).

*Cluster 4* contains the respondents who have declared to *totally agree* with the statement that they have the necessary skills for the job, but also *totally agree* that the robots steal jobs. Like the above two clusters, this group also declared itself to be an intensive Internet user. The most frequent category chosen by the respondents with respect to the item ‘Internet use’ was 4 (every day). The same category 4 (totally agree) was preferred by 100% of the respondents grouped in this cluster regarding the impact of robots on workplaces. 60.1% of the people included in this cluster chose category 4 (totally agree) on the item that refers to digital skills to do job (Fig 6).

We named this group the ‘*Internet users apparently very confident in their own digital skills*, *but also very fearful about the future of jobs in the context of digitalisation* (Fig 6).

*Cluster 2* is the smallest in terms of the number of the respondents. Like the people included in the previous clusters, they declare themselves Internet users, but they *tend to disagree* with the claim that are sufficiently skilled in the use of digital technologies to do the job. As expected, these people *totally agree* that robots are stealing jobs. The most frequent category of the ‘Internet use’ item was 4 (every day), chosen by 37.7% of the respondents in this cluster. Also category 4 (totally agree) was chosen by most of the respondents of this cluster to the item ‘robots steal jobs’ (52.2% of the total). At the item ‘digital skills to do their jobs’ the most frequent category was 2 (tend to disagree), preferred by 41% of the respondents of this cluster. This cluster can be seen as ‘*Internet users with low digital skills*, *very fearful about the impact of robots on the labour market*’ (Fig 6).

It is interesting to analyse the composition of the clusters from the point of view of the social categories of the respondents. Thus, in *cluster number 1*, women have a higher share. The level of education is high and the age group with the highest frequency of occurrence is 40–54 years. As regards the occupations of the respondents, most of them are manual workers, closely followed by other white collars and managers. As for the difficulty of paying bills, most of the respondents of this cluster stated that they have never had such difficulties.

**Fig 7:**
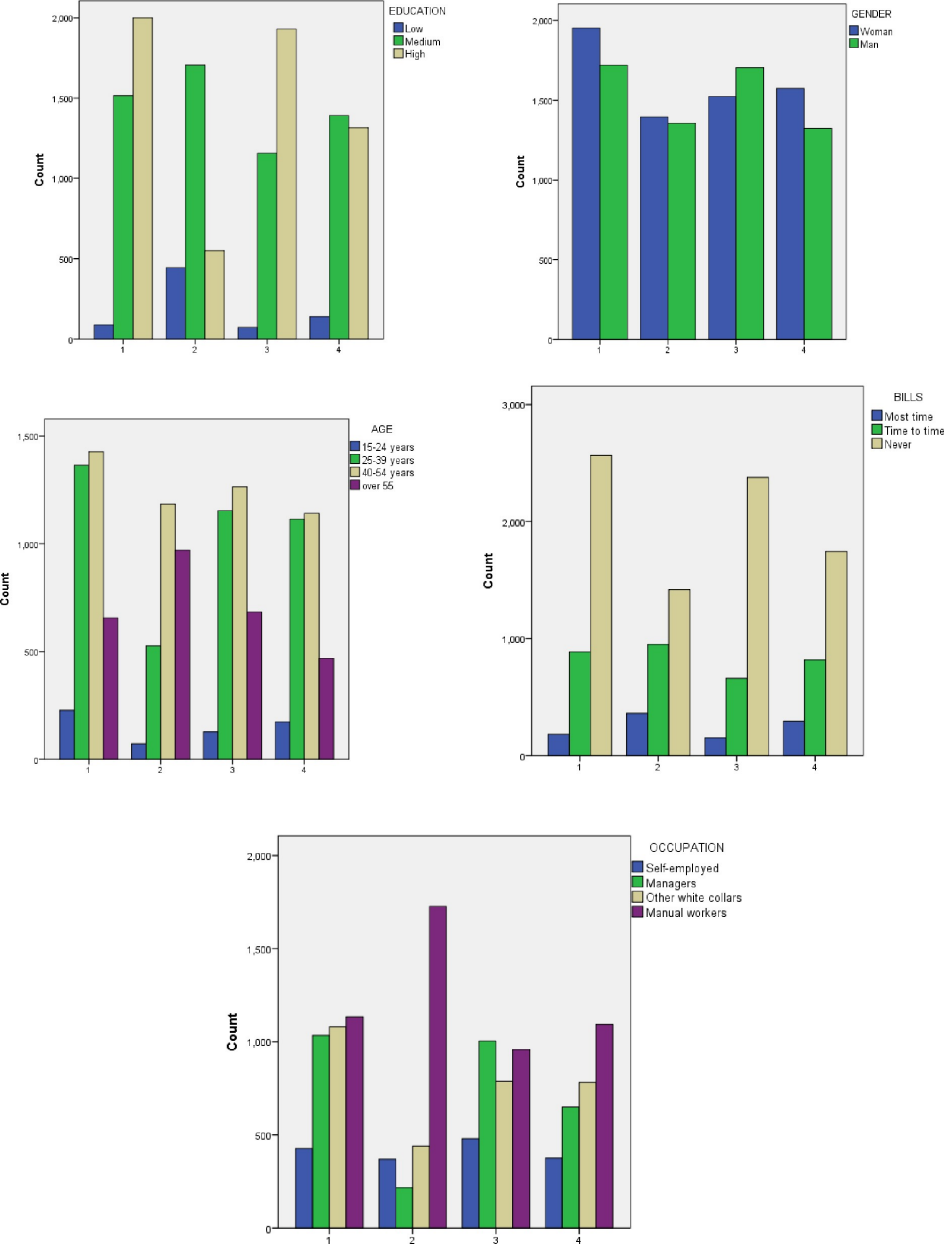
Social composition of the four clusters. Source: Authors' computations [40], using IBM SPSS Statistics 21.

*Cluster number 3* is composed of more men than women. The predominant level of education is the higher one and the age of most respondents is between 40–54 years. As regards respondents’ occupations, the highest frequency is of the managers. This cluster includes the smallest number of respondents who said they had difficulties paying bills most time. In *cluster number 4* more women than men were grouped. The predominant level of education is the average one and the most frequent age groups are 25–39 years and 40–54 years. This cluster is dominated by manual workers. *Cluster number 2* is the most balanced in terms of gender distribution, but very different from other clusters in terms of education level. The medium level of education predominates, but a high frequency of occurrence has the low level of education. From the point of view of age distribution, in this cluster were included mainly the persons in categories 40–54 years, respectively over 55 years. In terms of occupations, the highest frequency is manual workers. Unlike the cluster number 3, in cluster number 2 are the most respondents who had difficulty paying the bills most time (Fig 7).

**Fig 8:**
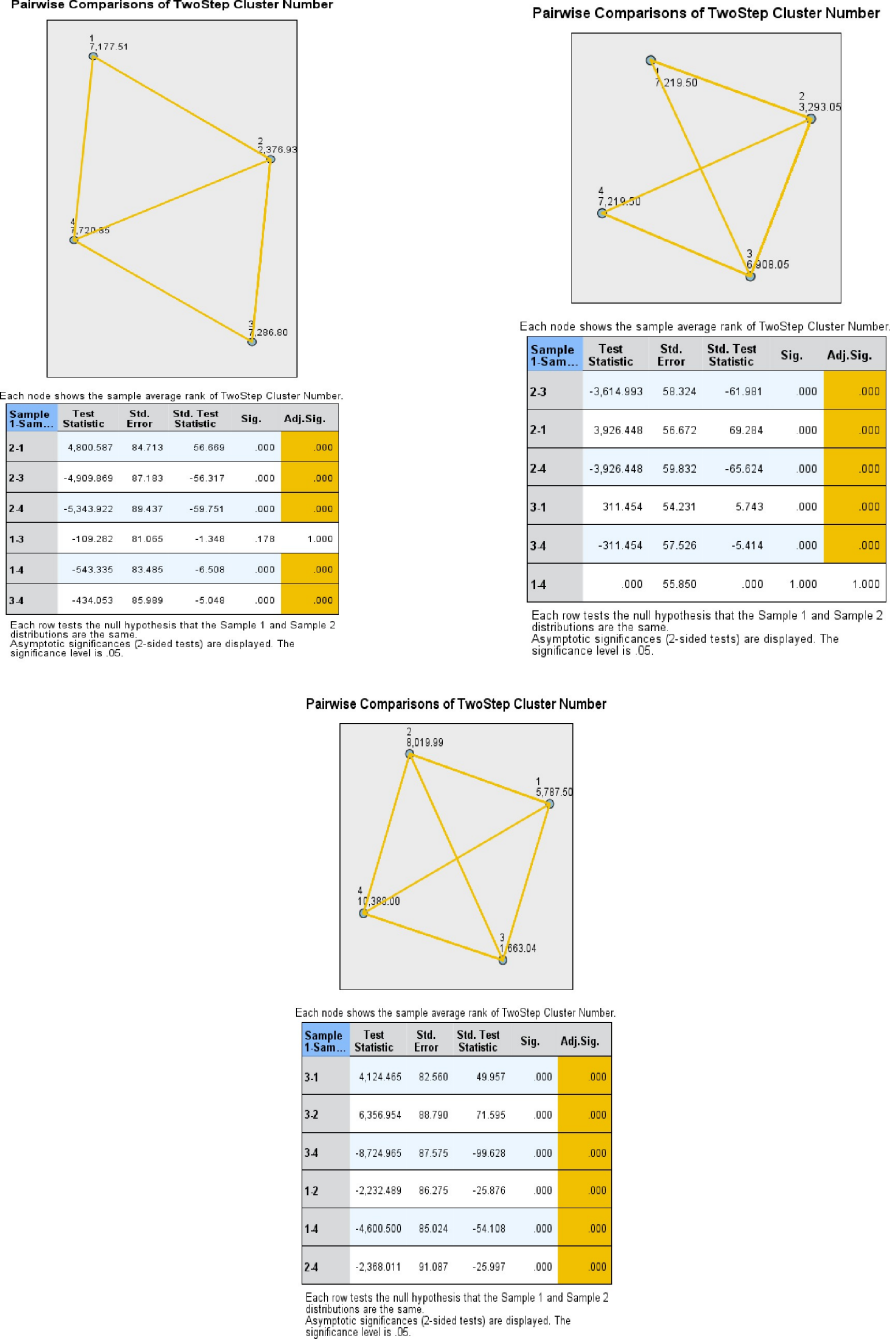
**Pairwise comparisons between clusters in terms of ‘digital skills’ (left), ‘robots steal jobs’ (middle) and ‘internet use’ (right).** Source: Authors' computations [40], using IBM SPSS Statistics 21.

Using Kruskal-Wallis test we have verified the independence of the clusters in relation to their components making pairwise comparisons. Thus, according to the pairwise comparisons, from the point of the item ‘digital skills to do job’ the most different clusters are cluster 2 and cluster 4, while the resembling ones are cluster 1 and cluster 3. In terms of the item ‘robots steal jobs’, all pairs of clusters proved to be independent, but the most different ones appeared to be cluster 3 and cluster 4, while somehow closer the pairs: cluster 1 and cluster 2, cluster 2 and cluster 4. As regards, the item ‘internet use’ all the pairs of clusters proved to be independent, except cluster 1 with cluster 4. The most different clusters seemed to be the pairs: cluster 2 and cluster 1, cluster 2 and cluster 4 (Fig 8).

Taking into consideration all these relationships, we have concluded that cluster 2 is the most different and, like the initial cluster 7, could be associated with the digital vulnerable groups and countries.

### 4.2. Logistic regression results

In order to test the H2-H4 we have used four logistic regression models. For testing the Hypothesis 2 *The effective use of these new technologies (in daily life as well as at work) depends on perceptions and skills*, *which in their turn are mainly determined by the level of education and income*, we have used two logistic regression models regarding people’s perception on skills needed in the use of digital technologies in daily life, as well as at work.

Table 5 contains the estimation results of the models. Both dependent variables were created by grouping the answers “totally agree” and “tend to agree” into value 1 and “tend to disagree” and “totally disagree” into value 0. The reference category is 0; therefore, the results will allow illustrating the profile of a digitally skilled respondent.

**Table 5:** Results of the logistic regression estimation–self assessment of skills in the use of digital technologies.

*Explanatory variables*		*Model 1*^a^			*Model 2*^a^		
		Digital tech skills: in daily life			Digital tech skills: to do job		
		B *(Coefficient)*	Wald test	Exp (B) *(Odds ratio)*	B *(Coefficient)*	Wald test	Exp (B) *(Odds ratio)*
*Intercept*		2.524*	67.287		2.690*	31.659	
Gender	Female	0.005	0.012	0.912	0.014	0.051	1.014
	Male (*ref*)	-	-	-	-	-	-
Age	15–24 years	0.792*	30.018	2.208	0.462*	8.208	1.587
	25–39 years	0.794*	111.409	2.212	0.434*	22.997	1.544
	40–54 years	0.377*	36.117	1.459	0.207*	6.528	1.230
	55 years and older (*ref*)	-	-	-	-	-	-
Education	Low (max 15 years of education)	-0.508*	42.910	0.602	-0.764*	37.561	0.466
	Medium (16–19 years of education)	-0.277*	25.404	0.758	-0.396*	28.584	0.673
	High (20 or more years of education) (*ref*)	-	-	-	-	-	-
Type of community	Rural area or village	0.042	0.491	1.043	-0.041	0.254	0.960
	Small/middle town	-0.050	0.755	0.951	-0.043	0.297	0.958
	Large town (*ref*)	-	-	-	-	-	-
Marital status	Single (or divorced or widow)	-0.039	0.604	0.437	0.061	0.723	1.063
	Married (or living with partner) *(ref)*	-	-	-	-	-	-
Occupation (Model 1)	Self-employed	0.366*	12.566	1.442	*NA*	*NA*	*NA*
	Employed	0.255*	20.718	1.291	*NA*	*NA*	*NA*
	Not working *(ref)*	-	-	-	*NA*	*NA*	*NA*
Occupation (Model 2)	Self-employed	*NA*	*NA*	*NA*	0.380*	15.088	1.463
	Managers	*NA*	*NA*	*NA*	0.847*	64.034	2.332
	Other white collars	*NA*	*NA*	*NA*	0.666*	63.562	1.946
	Manual workers *(ref)*	-	-	-	-	-	-
Difficulties paying bills	Most of the time	-0.008	0.010	0.919	-0.252*	5.305	0.777
	From time to time	0.011	0.037	0.847	-0.103	2.111	0.902
	Almost never/never *(ref)*	-	-	-	-	-	-
Social class	Working class of society	-0.264	0.729	0.393	-0.579	1.495	0.221
	Lower middle class of society	-0.180	0.336	0.562	-0.273	0.330	0.761
	Middle class of society	0.014	0.002	0.963	-0.218	0.214	0.644
	Upper middle class of society	0.187	0.339	0.560	0.070	0.020	1.072
	Higher class of society *(ref)*	-	-	-	-	-	-
Internet use	Never	-2.566*	1561.81	0.077	-1.957*	273.54	0.141
	Few times per month or less often	-1.462*	277.956	0.232	-1.248*	74.761	0.287
	Few times per week	-0.893*	148.718	0.410	-0.854*	70.712	0.426
	Every day *(ref)*	-	-	-	-	-	-
Impact in economy	Negative impact	-0.369*	24.926	0.691	-0.366*	13.499	0.693
	Positive impact *(ref)*	-	-	-	-	-	-
Impact in society	Negative impact	-0.270*	15.627	0.763	-0.225*	6.176	0.799
	Positive impact *(ref)*	-	-	-	-	-	-
Impact in daily life	Negative impact	-0.740*	115.482	0.477	-0.495*	26.645	0.610
	Positive impact *(ref)*	-	-	-	-	-	-
Robots opinion	Negative impact	-0.516*	105.679	0.597	-0.672*	100.134	0.510
	Positive impact *(ref)*	-	-	-	-	-	-
Read AI	No	-0.364	54.795	0.695	-0.518*	60.556	0.596
	Yes (*ref*)	-	-	-	-	-	-
Number of observations		17645			10040		
Pseudo R-Square (Cox and Snell)		0.314			0.186		
Pseudo R-Square (Nagelkerke)		0.473			0.315		

a. The dependent variable is a binary variable for which 1 = the respondent has the appropriate digital skills (auto evaluation)

* The parameter is statistically significant at 5%

Source: Authors’ estimation using IBM SPSS Statistics 21 [51]

#### Model 1

The first model was designed in order to analyse people’s digital skills used in daily life. The general form of model 1 is:

$$\begin{array}{l} {digitally\_skilled\_life}_{i}\\ \;=\beta_{0}+\beta_{1}∙gender+\beta_{2}∙age+\beta_{3}∙education+\beta_{4}∙community+\beta_{5}\\ \;∙marital\_status+\beta_{6}∙occupation+\beta_{7}∙bills+\beta_{8}∙social\_class+\beta_{9}\\ \;∙internet\_use+\beta_{10}∙impact\_economy+\beta_{11}∙impact\_society+\beta_{12}∙impact\_life\\ \;+\beta_{13}∙robots\_opinion+\beta_{14}∙read\_AI+\varepsilon_{i} \end{array}$$ (4.1.)

where the dependent variable is a binary variable that takes the value 1 if the respondent *totally agrees* or *tends to agree* that he/she is sufficiently skilled in the use of digital technologies in daily life.

The results indicate that gender is not statistically significant, meaning that there are no larger differences between men and women in regards to their everyday digital tech skills. But age matters–the obtained coefficients are statistically significant for all age groups and indicate that all the considered respondents are more digitally skilled than those 55 years or older (the reference category). Specifically, young people (15–24 years old) and young adults (25–39 years) are 2.2 times more likely to possess the digital skills needed in their daily lives compared to those over 55 years old. Also, those between the ages of 40 and 54 years are 1.4 times more likely to be proficient in digital technologies compared to those over 55. Thus, it can be seen that age plays an important role in people's ability to use the latest digital technology, age inequalities being significant.

Education also has a significant impact, the individuals with a low level of education (maximum 15 years) and those with a medium level (between 16 and 19 years of education) are less skilled in the use of digital technologies in everyday life, compared to people with higher education.

Next, we can point out that the type of community a person lives in, the marital status, the social class or the difficulties in paying the bills have no significant impact on one’s ability to use digital tech in daily life.

Regarding occupation, the results indicated that the self-employed are 1.4 times more likely to be sufficiently skilled in using digital technologies in daily life, compared to those not working, and, similarly, the employed have 1.3 times more chances to be digitally proficient.

After considering the classical socio-demographic variables, we extended the analysis by including some variables specific to the theme of digital technology: internet use, perception on the impact of technology development on the economy, society and on the quality of life, the general opinion of the respondents about robots, as well as a binary variable that takes the value 1 if the respondent has read about artificial intelligence in the last 12 months. Except reading about artificial intelligence, all the other considered variables were statistically significant.

The frequency with which a person uses the Internet has a significant impact on digital tech skills—compared to those who use the Internet daily, people who never use it have a 13 times lower chance of having the skills needed to use digital technologies in their daily lives; also, those who use the Internet a few times a month or less often, as well as those who use it a few times a week are less likely to be sufficiently skilled compared to those who use the Internet daily.

A good opinion about digital technology and robots has a positive impact on skills. Those who have said that recent technological developments have a positive impact on society, on the economy and increase the quality of life are more likely to have good digital skills, compared to those who perceive technological progress as having a negative effect.

#### Model 2

The second estimated model focused on people’s skills in the use of digital technologies to do their job. The general form of model 2 is:

$$\begin{array}{l} {digitally\_skilled\_work}_{i}\\ \;=\beta_{0}+\beta_{1}∙gender+\beta_{2}∙age+\beta_{3}∙education+\beta_{4}∙community+\beta_{5}\\ \;∙marital\_status+\beta_{6}∙occupation+\beta_{7}∙bills+\beta_{8}∙social\_class+\beta_{9}\\ \;∙internet\_use+\beta_{10}∙impact\_economy+\beta_{11}∙impact\_society+\beta_{12}∙impact\_life\\ \;+\beta_{13}∙robots\_opinion+\beta_{14}∙read\_AI+\varepsilon_{i} \end{array}$$ (4.2)

where the dependent variable is a binary variable that takes the value 1 if the respondent *totally agrees* or *tends to agree* that he/she is sufficiently skilled in the use of digital technologies to do their job

Once again, we see no gender disparities in this regard, both men and women having similar skills level. Age, on the other hand, is a significant factor influencing the digital skills required for work-related activities. Young people are the most skilled ones, the chances of being sufficiently skilled, decreasing with age. The inequalities determined by age are not as great as for the skills used in daily life; however, we see that young people are 1.4 times more likely to be sufficiently skilled in using digital tech at work compared to those aged 55 or older.

Taking into account the education variable, the results indicated that highly educated people are the best performers on the labour market in terms of using digital technologies, followed by those with a medium level of education and finally by those with a relatively low level of education (maximum 15 years of schooling). The differences are significant, those with higher education being 2.1 times more likely to be sufficiently skilled in using digital technologies at work compared to those with a maximum of 15 years of education.

The results of the logistic regression indicated that there are no significant differences in terms of competences depending on the social class, marital status or type of community.

For analysing occupations, we grouped the employed into four distinct groups: self-employed, managers, other white collars and manual workers. It has been observed that manual workers are the least skilled compared to other categories. Moreover, the differences are quite substantial: managers are 2.3 times more likely to be sufficiently skilled in using digital tech at work, whereas the other white collars have chances almost 2 times higher than manual workers to possess the necessary digital skills to do their job.

Unlike the previous model, we observe that for the digital skills used at work, there are significant differences between people with different levels of income. Using the difficulties in paying bills, as a proxy variable for income, the results indicated that those who do not face such difficulties are more likely to be sufficiently skilled in using digital tech at work compared to those facing difficulties in paying bills most of the time.

People that never use the Internet have seven times fewer chances to be sufficiently skilled in using digital technologies at work compared to individuals that use the Internet daily. As the frequency of Internet use increases, the differences with daily users become smaller. Given that for all the categories considered the results obtained were statistically significant, we can conclude that the frequency of Internet use is an essential influence factor of digital skills.

Once again, the positive perception of the effects of technology on society, the economy and the quality of life, as well as the generally positive perception about robots, proved to be significant factors contributing to a relatively higher level of digital skills. Moreover, it can be noticed that those who read about artificial intelligence are 1.7 times more likely to be sufficiently skilled to do their digital tech activities at work.

In order to test Hypotheses 3 and 4 (H3 and H4) we have used two models of logistic regression, estimated to observe people's perception on the impact of robots on the labour market and on their jobs. Table 6 summarises the results of the models.

**Table 6:** Results of logistic regression estimation impact of robots on future labour market.

Explanatory variables		Model 3^a^			Model 4^b^		
		The job can be done by a robot			Robots steal jobs		
		B *(Coefficient)*	Wald test	Exp (B) *(Odds ratio)*	B *(Coefficient)*	Wald test	Exp (B) *(Odds ratio)*
*Intercept*		0.199	0.785		-0.066	0.083	
Gender	Female	-0.154*	13.698	0.857	0.174*	15.082	1.190
	Male (*ref*)	-	-	-	-	-	-
Age	15–24 years	0.470*	18.610	1.600	-0.106	0.952	0.899
	25–39 years	0.375*	39.465	1.455	-0.047	0.578	0.954
	40–54 years	0.208*	13.591	1.232	-0.025	0.181	0.975
	55 years and older (*ref*)	-	-	-	-	-	-
Education	Low (max 15 years of education)	0.290*	8.310	1.336	0.641*	29.864	1.899
	Medium (16–19 years of education)	0.160*	11.468	1.174	0.243*	24.494	1.275
	High (20 or more years of education) (*ref*)	-	-	-	-	-	-
Type of community	Rural area or village	-0.255*	23.028	0.775	0.023	0.167	1.023
	Small/middle town	-0.177*	12.397	0.838	-0.117*	5.015	0.889
	Large town (*ref*)	-	-	-	-	-	-
Marital status	Single (or divorced or widow)	-0.106*	4.965	0.899	0.001	0.000	1.001
	Married (or living with partner) *(ref)*	-	-	-	-	-	-
Occupation	Self-employed	-0.394*	33.378	0.675	-0.251*	6.174	0.778
	Managers	-0.360*	35.387	0.697	-0.275*	8.780	0.760
	Other white collars	-0.034	0.393	0.966	-0.081	0.807	0.922
	Manual workers *(ref for model 3)*	-	-	-	-0.084	0.963	0.919
	House persons	*NA*	*NA*	*NA*	0.001	0.000	1.001
	Unemployed *(ref for model 4)*	*NA*	*NA*	*NA*	-	-	-
Difficulties paying bills	Most of the time	0.193*	5.487	1.213	0.264*	8.918	1.302
	From time to time	0.276*	31.475	1.318	0.190*	13.121	1.210
	Almost never/never *(ref)*	-	-	-	-	-	-
Social class	Working class of society	0.052	0.055	1.053	0.971*	20.762	2.641
	Lower middle class of society	0.074	0.111	1.077	0.664*	9.734	1.943
	Middle class of society	-0.045	0.043	0.956	0.672*	10.585	1.959
	Upper middle class of society	-0.003	0.000	0.997	0.183	0.729	1.201
	Higher class of society *(ref)*	-	-	-	-	-	-
Internet use	Never	0.048	0.219	1.049	0.193**	2.764	1.212
	Few times per month or less often	0.236**	3.139	1.266	0.245	2.659	1.278
	Few times per week	0.112	1.537	1.118	0.112	1.275	1.118
	Every day *(ref)*	-	-	-	-	-	-
Impact in economy	Negative impact	0.113	2.002	1.119	-0.169*	3.847	0.845
	Positive impact *(ref)*	-	-	-	-	-	-
Impact in society	Negative impact	-0.125*	3.913	0.882	0.116**	2.852	1.123
	Positive impact *(ref)*	-	-	-	-	-	-
Impact in daily life	Negative impact	-0.040	0.301	0.961	-0.027	0.119	0.973
	Positive impact *(ref)*	-	-	-	-	-	-
Robots opinion	Negative impact	-0.711*	189.092	0.491	0.882*	213.438	2.417
	Positive impact *(ref)*	-	-	-	-	-	-
Read AI	No	-0.164*	13.650	0.848	0.248*	28.004	1.281
	Yes (*ref*)	-	-	-	-	-	-
Number of observations		10077			12335		
Pseudo R-Square (Cox and Snell)		0.049			0.077		
Pseudo R-Square (Nagelkerke)		0.065			0.113		

a. The dependent variable is a binary variable for which 1 = the respondent agrees that his current job can be done by a robot (totally or partially)

b. The dependent variable is a binary variable for which 1 = the respondent agrees that robots and artificial intelligence steal people’s jobs

* The parameter is statistically significant at 5%

** The parameter is statistically significant at 10%

Source: Authors’ estimation using IBM SPSS Statistics 21 [51]

#### Model 3

The general form of model 3 is:

$$\begin{array}{l} {job\_done\_by\_robots}_{i}\\ \;=\beta_{0}+\beta_{1}∙gender+\beta_{2}∙age+\beta_{3}∙education+\beta_{4}∙community+\beta_{5}\\ \;∙marital\_status+\beta_{6}∙occupation+\beta_{7}∙bills+\beta_{8}∙social\_class+\beta_{9}\\ \;∙internet\_use+\beta_{10}∙impact\_economy+\beta_{11}∙impact\_society+\beta_{12}∙impact\_life\\ \;+\beta_{13}∙robots\_opinion+\beta_{14}∙read\_AI+\varepsilon_{i} \end{array}$$ (4.3)

where the dependent variable is a binary variable that takes the value 1 if the respondent *totally agrees* or *tends to agree* that his/her current job could be done by a robot or by artificial intelligence.

It is important to note that this time there are gender disparities, as women are less likely to think that their work can be done by a robot. We also face age-related inequalities, for all age groups obtaining statistically significant coefficients. The positive results indicate that all age groups are more likely to consider that robots can replace them in the workplace compared to those aged 55 and over, the highest differences being registered for young people. As usual, education plays a vital role in people's perception of the involvement of robots in the labour market. Those with a low level of education are 1.3 times more likely to consider that they will be replaced by robots, compared to those with higher education. Also, high school graduates are more likely to believe this.

Within this model, we obtained that perceptions differ depending on the type of community. Both those in rural areas and those in small and medium-sized cities are less likely to consider that they can be replaced by robots at work, compared to people living in large towns. It is also interesting that single (or divorced, or widowed) people are less likely to think that their work can be done by a robot, compared to the married people (or those living with a partner). Regarding occupation, the results indicated that self-employed, as well as managers, are less likely to consider that they can be totally or partially replaced at work by a robot, compared to manual workers.

The level of income seems to be relevant, those who have difficulty paying bills most of the time or from time to time have higher chances of considering that robots can do their job, compared to those who have never had difficulty paying bills. On the other hand, the social class did not prove to be statistically significant.

Regarding the variables specific to digital technology, the results are no longer as uniform as in the previous models. Significant differences turned out to be only between those who use the Internet few times a month or less often compared to those who use it daily, indicating that daily Internet users are less likely to be replaced by robots at work. Also, the positive perception regarding the impact of digital technologies on the economy and on the quality of life is no longer significant. But, those with positive perception about robots, the impact of technology on society and those who read about artificial intelligence are more likely to believe that their job can be done by a robot—a result that may indicate a form of awareness that technological changes will have an impact on jobs.

On the other hand, the fact that people think they can be replaced by robots may indicate that they cannot easily adapt to new technologies. Indeed, there are certain types of work that can be automated, but this should not necessarily leave people without jobs, but instead cause the transformation of the labour market. Technological progress, which is imminent, will redefine jobs rather than eliminating them. Based on model 3 we aimed to see which categories of people consider robots as a substitute, these being the ones that have the most considerable difficulties in adapting to digitalisation, in obtaining or rapidly completing their skills according to the new technologies. Thus, the results of the logistic regression pointed out that the most vulnerable categories in the workplace in the face of new technologies are: men, people with a low level of education, those living in large towns, married people, manual workers, and people with low income.

#### Model 4

The last logistic regression model focused on estimating the critical factors that influence one’s opinion that robots steal people’s jobs. The general form of model 4 is:

$$\begin{array}{l} {robots\_steal\_jobs}_{i}\\ \;=\beta_{0}+\beta_{1}∙gender+\beta_{2}∙age+\beta_{3}∙education+\beta_{4}∙community+\beta_{5}\\ \;∙marital\_status+\beta_{6}∙occupation+\beta_{7}∙bills+\beta_{8}∙social\_class+\beta_{9}\\ \;∙internet\_use+\beta_{10}∙impact\_economy+\beta_{11}∙impact\_society+\beta_{12}∙impact\_life\\ \;+\beta_{13}∙robots\_opinion+\beta_{14}∙read\_AI+\varepsilon_{i} \end{array}$$ (4.4)

where the dependent variable is a binary variable that takes the value 1 if the respondent *totally agrees* or *tends to agree* that robots and artificial intelligence steal peoples' jobs.

The results indicate that gender inequalities manifest significantly in this situation, with men being more likely to fear that robots will leave people without jobs. An exciting result comes concerning age, as it no longer is statistically significant, meaning that between different age groups, there are similar beliefs.

Education is still a determinant of inequalities, the most constant in all analysis. Those with a low level of education are most likely to fear that robots will steal people's jobs, thus indicating their vulnerability to accelerated technological progress. Those with a medium level of education also show fear in front of the involvement of robots and artificial intelligence in the labour market, thus being more disadvantaged compared to those with higher education.

The marital status has no significant influence on people’s perception, and when it comes to community type, we noticed a slight decrease of chances for those living in small or medium towns to believe that robots steal people’s jobs, compared to individuals living in large cities.

The analysis on occupations used the unemployed as a reference category. The results pointed out that the self-employed and the managers are less likely to consider that robots and artificial intelligence steal people’s jobs, compared to the unemployed. All other viewed categories were not statistically significant.

The fear that robots will steal people's jobs is more pronounced in the case of people who have difficulty paying bills, either most of the time or only from time to time, compared to those who do not have such financial problems. Also, we obtained significant differences depending on the social class. Individuals belonging to the working class of society are 2.6 times more likely to fear that robots steal people’s jobs, compared to those in the higher class of society. Similar results were obtained for those in the lower middle class and in the middle class of the society, in comparison with those belonging to the higher class.

Regarding Internet use, those who never use the Internet are more likely to consider that robots steal people's jobs compared to daily users. The same is true for those who read books about artificial intelligence, compared to those who haven't done so in the past 12 months.

An interesting aspect highlighted by our analysis was that individuals with a positive general opinion about robots have a 2.4 times higher chance of considering that robots steal people's jobs. On the contrary, those who believe that digital technologies have a positive impact on the economy have a lower chance of understanding that robots will leave people without their jobs.

### 4.3. The people's attitude towards digitalisation in EU—The need for a smart labour market

**Fig 9:**
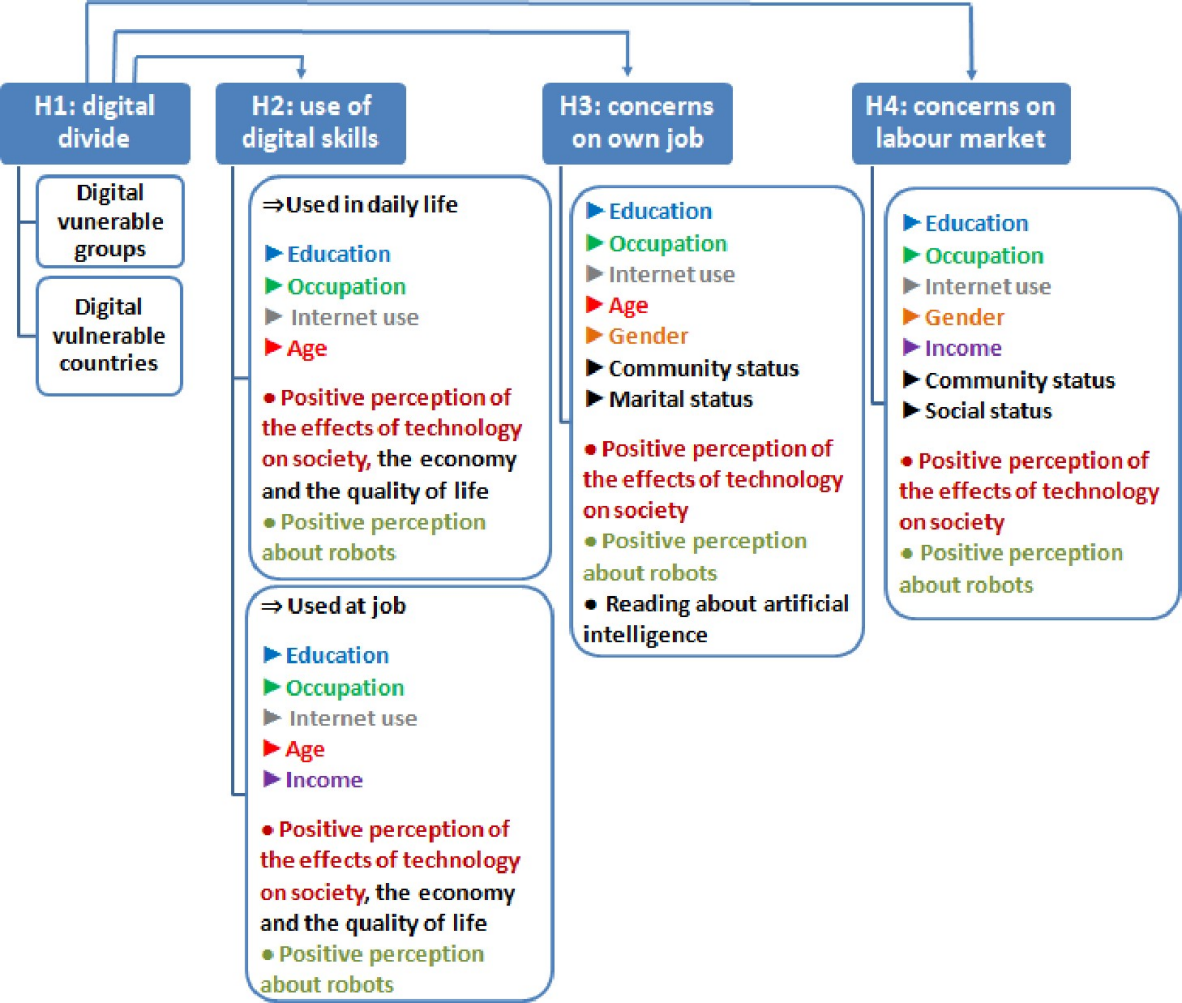
The research hypotheses and results.

Our research aimed to identify factors with high impact on creating vulnerable groups (of people or countries) and to highlight the more likely variables to create disparities in terms of skills and attitude towards digitalisation. Our objective was to find relevant variables for labour market in order to assume targeted measures that should be taken for a better match of labour supply and demand and for creating a smart labour market in order to increase the people's confidence in their skills level and to make the most of digitalisation of the societies. Fig 9 summaries the influencing factors resulted from our empirical analysis.

The cluster analysis we performed confirmed the H1: *Digital divide could lead to the creation of ‘digital vulnerable groups’ and more than that of ‘digital vulnerable countries’*. One of the final four clusters, namely cluster number 2 has been identified with the ‘digital vulnerable group’ of citizens. It proved to be the most different compared to the others (but similar to cluster number 7 from the initial solution): with 73% of the respondents having low or very low digital skills, 95% being afraid or very afraid that robots could steal their jobs and 26% with low usage of the internet. Almost 80% of the respondents in cluster 2 have a low or medium level of education, 51% are women, nearly 80% are over 40 years old, 63% are manual workers, and almost a half have problems in terms of paying bills. These are the ‘digital vulnerable social categories’. Four countries (Hungary, Greece, Romania and Bulgaria) could be seen as the most ‘digital vulnerable countries’, as they have large shares of the citizens that grouped in cluster 2: between 34% and 42% of the respondents.

'Digital vulnerable countries' tend to be, as expected, low-income countries (countries with GDP below the EU average, e.g. Hungary 62.4% of EU average, Greece 82.4% of EU average, Romania 50.4% EU average and Bulgaria 49.5% EU average [52]).

Highly developed countries (countries with GDP over the EU average: the Netherlands 111.8% of EU average, Denmark 132.2% of EU average, Finland 124% of EU average, Sweden 130% of EU average, the United Kingdom 111% of EU average) are less vulnerable to digitalisation impact, and their citizens are less fearful about the effects of automation. Their citizens use the internet every day (94.9% of the respondents), mostly they do not fear that robots steal their jobs, and they consider having the necessary digital skills to do their job.

We have found for both models used for testing the H2 (*The effective use of these new technologies (in daily life as well as at work) depends on perceptions and skills*, *which in their turn are mainly determined by the level of education and income*) that a positive attitude towards digital technology and robots and also towards the effects of technology on society, the economy and the quality of life are important factors contributing to an increased level of digital skills for using technologies in daily life and at work. The level of education has a significant impact on the level of digital skills both in everyday life and at work, but it proved to be more important for the skills needed at work. In the second case (at work) the differences are more pronounced, citizens with higher education being 2.1 times more likely to be sufficiently skilled in using digital technologies at work compared to those with a relatively low level of education. A difference between the two models was observed in terms of income level. The income has no significant impact on people’s ability to use digital technologies in daily life, but for developing the necessary skills to do their job, income level proved to be a significant factor. Considering these, the H2 was confirmed for people using digital skills at work, and it was partly confirmed for people using digital skills in their lives.

Model 3 confirmed the H3 *The general perception of EU citizens about the digitalisation is a positive one*, *but there are some categories that feel insufficiently prepared for the assimilation of new technologies*, *especially in their workplace*. Even if the general perception regarding the impact of digital technologies on the economy and on the quality of life is mostly positive, we didn’t find a statistically significant relationship with the concerns regarding the impact on the labour market. But factors like reading about artificial intelligence, positive perception about robots, the impact of technology on society proved to be significant for rising concerns regarding that current jobs could be done by robots and artificial intelligence. Also, the logistic regression showed other significant factors influencing these concerns: age, gender, education, community type, occupation and marital status, as well as income level.

Model 4 confirmed the H4 *People’s perception of robots is generally a positive one*, *but there are major concerns*, *especially regarding the impact of digitalisation on the labour market and jobs*. The general attitude towards robots is a positive one, and the results indicated that people with that positive attitude are more likely to believe that robots steal people's jobs. Overall, the results showed that people who have greater contact with the digital world (use the Internet every day, read about artificial intelligence) are more aware of the changes that will occur in the labour market, namely that some of the current work will be made by robots in the near future Also, the logistic regression showed that several socio-demographic characteristics significantly influence this concern: gender, education, social class, social status, occupation and income. The socio-demographic profile of a person who fears that robots will steal people's jobs is most likely female, with a low level of education, unemployed and with financial difficulties.

The digitalisation and the effects of technologies on economy, society and quality of life imply major challenges on the labour market. As innovation and technological progress are faster than both the changes in people's perception on technologies' impact as well as their digital skills, it is necessary to address the challenges that digitalisation brings to the labour market by appropriate measures in order to successfully change the manner of doing business, working and living. The results of our research highlight the importance of public and private involvement for better connectivity increased confidence and fewer concerns for citizens regarding the impact of digitalisation on their lives and on socio-economic environment as a whole. The labour market will transform, integrating the effects of digitalisation, becoming smarter. From the new challenges faced by the labour market on its way of becoming smart, having digitalisation as driving force, we consider to be of great importance:

- the need of improving the lifelong learning processes to continually adapt the skills supply to the need of the economy, reshaped by the impact of digitalisation of processes and automation of work (addressing the digital skills gap);
- the demographic changes and resulted need to adapt to an ageing population with specific socio-economic characteristics and perceptions on innovation and digitalisation—the elderly represent a segment targeted by digital technologies aimed at improving their quality of life, but it is necessary to pay special attention to the abilities of these people to use these technologies;
- changes in occupational structure because many jobs will change or disappear, and many others will be substituted or created. Education is playing an increasing role as many people with a low level of education, and poor qualification will have to be relocated to tasks that are not susceptible to be performed by robots or artificial intelligence. These changes should be carefully managed to reduce the risk of increasing inequality and polarization within society.

As digitalisation will have a meaningful impact on the economy and society in the EU in next period, the positive attitude towards digitalisation is essential for transforming this relatively new challenge into an excellent opportunity for the future.

## V. Concluding remarks

Digitalisation has an increasing impact on many aspects of the economy and society in the EU, both on public (government) or private sectors (banking, commerce, etc.). Under these circumstances, the digital skills endowment of the population represents a critical issue to succeed in different fields affected by the digitalisation and consequently on the labour market.

The analysis of EU citizens' attitude towards digitalisation shows that digitalisation and automation are primarily treated as opportunities. Also, if the citizens i) are using technologies (at work or in daily life) being endowed with necessary digital skills and ii) are more informed about these technologies, the attitude/ perception is positive, and they trust the new technologies.

Starting from the objective of our research we have analysed if digital divide leads to the creation of vulnerable citizens or countries groups and if perception patterns exist, focusing on skilled used in daily life and at work and on effects of digitalisation on the labour market and people's lives.

TwoStep Cluster Analysis enabled us to highlight the homogeneous groups of EU citizens in terms of their attitudes towards digitalisation, the abilities to use ICT at work and the actual use of the Internet. As we have assumed from the beginning, there are some social groups more ‘digital vulnerable’ than others and some countries with a population more fearful about robots stealing jobs than the citizens of other countries. Women are more afraid than men of what the future can bring to them in terms of the workplace digitalisation. Besides, people over 55 years, with a low level of education (mainly manual workers) and a low standard of living form the most vulnerable category in digitalisation era. The latter citizens come from countries such as Hungary, Greece, Romania and Bulgaria.

The logistic regression models allowed us to analyse the people’s perception on being sufficiently skilled in the use of digital technologies in daily life or at work and the people's perception on the impact of robots on their jobs and on the labour market, considering as explanatory variables variable the citizens' socio-demographic characteristics and the people's perception on effects of digitalisation on the economy, society, quality of life.

We found that people who feel confident they have the necessary level of digital skills for daily life or job have a positive perception regarding the effects of technologies on economy, society, quality of life and positive perception about robots. Concerning the socio-economic characteristics, they are highly skilled (education), employed or self-employed (occupation), young adults (age) and have frequent access to the Internet. Even the respondents who believe that robots can do their jobs or that the robots will steal their jobs are persons who have a positive perception with regards to the effects of technologies on society and also a positive perception about robots. In terms of occupation, the self-employed and the managers are less likely to consider that robots can take their job or can replace them on the labour market. Other socio-demographic characteristics proved to be essential for people's perception (fear) on the impact of robots: education (persons with a low level of education), gender (women), income (individuals with financial difficulties) and internet use (those who never use the Internet).

Our analysis made it possible to identify a general profile of vulnerable people in the face of digitalisation. In essence, they are most likely to be elderly, with a low level of education, manual workers or not working, with a relatively low level of income and little Internet use.

One of the limitations of this study is that we only analyse people's perceptions, which, by definition, are subjective, so that the actual level of digital skills cannot be quantified. Also, perceptions can be influenced by the fact that people do not know/understand very well what robots or artificial intelligence entail and are fearful for precisely this reason, not necessarily because they could not adapt to changes in the labour market.

The results of our analysis highlighted that public and private actors should develop concrete and targeted measures (better regulations/control and investments in high-quality education and lifelong learning process) to reduce people fears and increase confidence in the safety of using new ICT and specific, country-oriented measures to reduce other economic and social inequalities in order to fill the ‘digital gaps’. This involvement should also be considered under the current demographic trends, and ageing population, which calls for quality education and lifelong learning process for a better correlation of skills supply with demand on the labour market.

We estimate that digitalisation will contribute decisively to defining the *new man*, in a different manner from that characterising the previous period, but tighter in control. The benefits in terms of improving the quality of life quality, diminishing generation gap (baby boomers, millennials or generation Z will have common points in it) and the impact on a green and sustainable future make from digitalisation a key input in the society as a whole.
